# Electrochemical generation of hydrogen peroxide from a zinc gallium oxide anode with dual active sites

**DOI:** 10.1038/s41467-023-37007-9

**Published:** 2023-04-05

**Authors:** Lejing Li, Zhuofeng Hu, Yongqiang Kang, Shiyu Cao, Liangpang Xu, Luo Yu, Lizhi Zhang, Jimmy C. Yu

**Affiliations:** 1 Department of Chemistry, The Chinese University of Hong Kong, Hong Kong, China; 2 School of Environmental Science and Engineering, Guangdong Provincial Key Laboratory of Environmental Pollution Control and Remediation Technology, Sun Yat-sen University, Guangzhou, 510275 China; 3 Institute of Materials Research, Tsinghua Shenzhen International Graduate School, Tsinghua University, Shenzhen, 518055 China; 4 Key Laboratory of Pesticide & Chemical Biology of Ministry of Education, Institute of Applied & Environmental Chemistry, College of Chemistry, Central China Normal University, Wuhan, 430079 China

**Keywords:** Electrocatalysis, Catalyst synthesis, Electrocatalysis

## Abstract

Electrochemical water oxidation enables the conversion of H_2_O to H_2_O_2_. It holds distinct advantages to the O_2_ reduction reaction, which is restricted by the inefficient mass transfer and limited solubility of O_2_ in aqueous media. Nonetheless, most reported anodes suffer from high overpotentials (usually >1000 mV) and low selectivity. Electrolysis at high overpotentials often causes serious decomposition of peroxides and leads to declined selectivity. Herein, we report a ZnGa_2_O_4_ anode with dual active sites to improve the selectivity and resist the decomposition of peroxides. Its faradaic efficiency reaches 82% at 2.3 V versus RHE for H_2_O_2_ generation through both direct (via OH^−^) and indirect (via HCO_3_^−^) pathways. The percarbonate is the critical species generated through the conversion of bicarbonate at Ga-Ga dual sites. The peroxy bond is stable on the surface of the ZnGa_2_O_4_ anode, significantly improving faradaic efficiency.

## Abstract (web summary)

Developing efficient anodes for H_2_O_2_ production via water oxidation remains challenging. Here, the authors report a ZnGa_2_O_4_ anode with dual active sites to improve the selectivity and to resist the decomposition of H_2_O_2_, and the peak faradaic efficiency reaches 82% at a low potential.

## Introduction

Hydrogen peroxide (H_2_O_2_) is a clean and valuable oxidant with wide applications in chemical synthesis^1,2^, energy technology^3–5^, and environmental remediation^6–8^. Currently, most H_2_O_2_ is produced by an anthraquinone process^9^, which requires expensive Pd catalysts and produces a huge volume of organic waste^10^. Considerable efforts have been dedicated to producing H_2_O_2_ in small-scale and more sustainable ways^11–14^. For example, the direct synthesis method (Eq. (1)) has been developed using H_2_, O_2_, and noble metal catalysts in a liquid medium^15–17^. However, inert gases (N_2_ or CO_2_) are needed to dilute the reactant gases to eliminate the explosive risk.

1 $${{{{{{\rm{H}}}}}}}_{2}+{{{{{{\rm{O}}}}}}}_{2}\to {{{{{{\rm{H}}}}}}}_{2}{{{{{{\rm{O}}}}}}}_{2}\,\triangle {{{{{\rm{G}}}}}}_{298{{{{\rm{K}}}}}}^{{{{{\rm{o}}}}}}{{\rm {=}}}-\!135{{{{\rm{.}}}}}9\,{{{{\rm{kJ}}}}}\,{{{{{\rm{mol}}}}}}^{-1}.$$

The inherent limitations of Eq. (1), including the low solubility and inefficient mass transfer of gas reactants, the requirement for high-valued H_2_, and the safety concerns, have motivated researchers toward more feasible production routes. Alternatively, H_2_O_2_ generation can start with abundant feedstocks such as water and oxygen. The main-stream O_2_ reduction reaction (ORR, Eq. (2)) has been demonstrated on some noble metals or carbon-based catalysts^12,18–20^.

2 $${{{{{{\rm{O}}}}}}}_{2}+{2{{{{{\rm{H}}}}}}}^{+}+{2{{{{{\rm{e}}}}}}}^{-}\rightleftharpoons {{{{{{\rm{H}}}}}}}_{2}{{{{{{\rm{O}}}}}}}_{2}\,{{{{{{\rm{E}}}}}}}^{0}=+ \!0.68{{{{{\rm{V}}}}}} \,{{{{{\rm{versus}}}}}}\, {{{{{\rm{RHE}}}}}}.$$

However, 2 electron-ORR is subjected to low solubility (8 mg L^−1^, 1 atm, 25 °C) and low diffusion coefficient of O_2_ in water (2.1 × 10^−5^ cm^−2^ s^−1^)^21^. The O_2_ reduction way requires continuous feeding of O_2_ or air to the cathode surface, which brings extra production costs. Though these mass transfer problems can be overcome by gas diffusion electrodes (GDEs), it has been widely recognized that the effectiveness of GDEs suffers from a common flooding effect^22,23^, i.e. electrolyte penetration into the porous GDE. By contrast, the kinetics of the water oxidation reaction (WOR, Eq. (3)) would not be limited by the mass transfers of reactants. A high faradaic current density can therefore be attained. 2e-WOR can meet the onsite demand for H_2_O_2_ in O_2_-deficient environments, such as bacteria-contaminated water bodies^8^, which represents an obvious advantage over other production methods that require O_2_. Besides, the WOR reaction can be coupled with reduction reactions at the cathode, including hydrogen evolution, ORR, and CO_2_ reduction reaction, to fully utilize the half-cell reactions.

3 $$2{{{{{{\rm{H}}}}}}}_{2}{{{{{\rm{O}}}}}}\rightleftharpoons {{{{{{\rm{H}}}}}}}_{2}{{{{{{\rm{O}}}}}}}_{2}+2{{{{{{\rm{H}}}}}}}^{+}+2{{{{{{\rm{e}}}}}}}^{-}\,{{{{{{\rm{E}}}}}}}^{0}=+ 1.76{{{{{\rm{V}}}}}}\, {{{{{\rm{versus}}}}}}\, {{{{{\rm{RHE}}}}}}.$$

There are still several challenges to use the 2e-WOR technique effectively. First, the faradaic efficiency (FE) of H_2_O_2_ is low, especially at low overpotentials. Though some metal oxides and carbon-based catalysts have been reported for 2e-WOR^7,24–26^, most of these catalysts usually require overpotentials larger than 1000 mV to achieve a satisfactory FE. High overpotentials required for anodes will reduce the overall energy efficiency of the electrocatalytic cells. Recently, progress has been made in lowering the overpotential by fabricating new anodes, for example, H_2_O_2_ FE reaching 66% on CP/PTFE with an overpotential of 640 mV^27^, and reaching 72% on CuWO_4_ with an overpotential of 740 mV^28^. Moreover, the electrochemical stability (including stable generation rate and FE) and the structural stability are also challenging because of the highly oxidative environment. Nevertheless, the main goal of 2e-WOR research is still to develop efficient and stable anodes with high selectivity at low overpotentials. For the studies of anodic H_2_O_2_ production, the performance of anodes is usually evaluated in bicarbonate electrolytes (usually 2 M KHCO_3_) since many studies have demonstrated the promotion effect^7,29–31^ and the stabilizing effect^32^ of bicarbonate toward anodic H_2_O_2_ production. A critical but often ignored issue is the decomposition of H_2_O_2_ during long-time water oxidation. For example, the H_2_O_2_ generation rate declines after passing 100 C for FTO/BiVO_4_^29^, after 3 h-electrolysis for FTO/CaSnO_3_^24^, after 3 h-reaction for FTO/Sb_2_O_3_^26^, after for 1000 s for boron-doped diamond^33^, and after 150 min-electrolysis for CaSnO3@CF^34^. A similar observation was also reported on C, N-codoped TiO_2_ in 0.05 M Na_2_SO_4_ after 2 h-electrolysis^35^. In these reports (details in Table S1), the H_2_O_2_ concentration reaches a saturation level and does not increase further. Factors associated with the concentration plateau are considered inseparable from the instability of H_2_O_2_ during electrolysis but await in-depth investigation. Typically, the decomposition of H_2_O_2_ can occur either on the anode surface or in the bulk electrolyte. Firstly, the oxidation of H_2_O_2_ occurs thermodynamically at a relatively low potential of 0.68 V versus RHE (reverse of Eq. (2)). Therefore, there is a need to develop anode materials with sluggish H_2_O_2_ oxidation kinetics. Otherwise, electrolysis at high potentials would cause severe H_2_O_2_ electro decomposition on the anode surface. Secondly, anode material may induce H_2_O_2_ decomposition, and the decomposition rate is material-dependent^26,30,36^. For instance, the current density associated with the electro decomposition of H_2_O_2_ on BiVO_4_ is estimated to be ~11 μA cm^−2^ mM H_2_O_2_ in 0.5 M KHCO_3_ electrolyte^30^, which is an order of magnitude smaller than that on Pt surface in phosphate buffer^36^. Thirdly, H_2_O_2_ can undergo disproportionation (Eq. (4)) to give rise to O_2_ and water with the release of heat.

4 $${{{{{{\rm{H}}}}}}}_{2}{{{{{{\rm{O}}}}}}}_{2}({{{{{\rm{l}}}}}})\to {{{{{{\rm{H}}}}}}}_{2}{{{{{\rm{O}}}}}}({{{{{\rm{l}}}}}})+\frac{1}{2}{{{{{{\rm{O}}}}}}}_{2}({{{{{\rm{g}}}}}})\,\Delta{{{{{{\rm{G}}}}}}}_{298\,{{{{{\rm{K}}}}}}}^{{{{{{\rm{o}}}}}}}=-\!116.7\,{{{{{\rm{kJ}}}}}}\,{{{{{{\rm{mol}}}}}}}^{-1}$$

Based on these considerations, one can anticipate that developing anodes with a high tolerance and low decomposition rate of H_2_O_2_ are prerequisites for achieving high FE.

Here, we develop a ZnGa_2_O_4_ anode featuring dual active site for H_2_O_2_ generation through WOR. At 2.3 V versus RHE, this ZnGa_2_O_4_ shows its highest FE of ~82%. Significantly, by combining the in situ spectral evidence and theoretical calculations, it is identified that the generation of H_2_O_2_ follows both the direct (OH^-^-mediated) and indirect (HCO_3_^-^-mediated) pathway at the ZnGa_2_O_4_ anode. Theoretical studies indicate that bicarbonate species can bind with Ga-Ga dual sites and then be oxidized to percarbonate species. The O–O bond in peroxide species is stable on the surface of ZnGa_2_O_4_, which helps to maintain high FE and high H_2_O_2_ concentration levels.

## Results

### Anode material characterizations

**Fig. 1:**
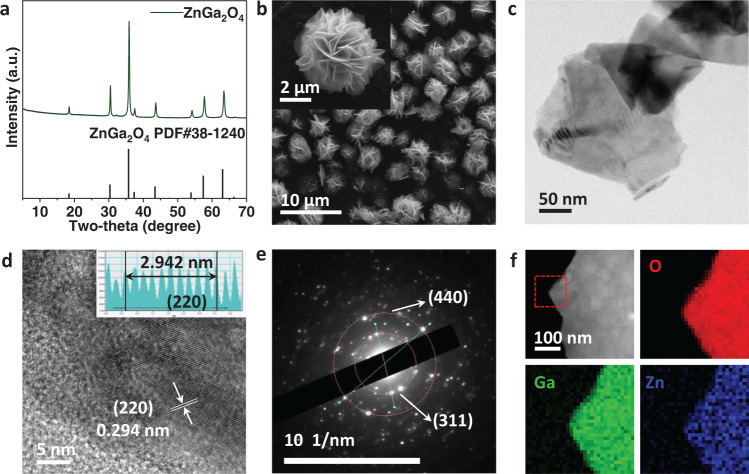
Morphological characterizations of ZnGa_2_O_4_. **a** X-ray diffraction pattern of the prepared ZnGa_2_O_4_. **b** SEM images of ZnGa_2_O_4_. **c** TEM image. **d** HRTEM image. **e** Polycrystalline electron diffraction pattern of ZnGa_2_O_4_. **f** Scanning transmission electron microscopy electron energy loss spectroscopy (STEM-EELS) of the ZnGa_2_O_4_.

The nanoflower-like ZnGa_2_O_4_ was synthesized by the solvothermal method modified from a reported procedure^37^. The X-ray diffraction pattern of as-prepared sample is in accordance with that of spinel ZnGa_2_O_4_ (PDF#38-1240) (Fig. 1a), indicating a pure ZnGa_2_O_4_ phase with high crystallinity. SEM image reveals the ZnGa_2_O_4_ catalyst has a monodisperse microsphere with a uniform diameter of ~5 μm (Fig. 1b). It is clearly shown a flower-like structure consisting of self-assembly nanosheets. From the transmission electron microscopy (TEM) images (Fig. 1c, d), the nanosheets show irregular shape and clear lattice fringes were observed in the high-resolution TEM image (Fig. 1d and Figure S1), indicating the high crystallinity of the catalysts. The lattice fringes in Fig. 1d spaced 0.294 nm apart can be well assigned to the (220) facet of ZnGa_2_O_4_, and the lattice fringes spaced 0.25 nm apart (Figure S1c, d) can be assigned to (311) facet. From the polycrystalline electron diffraction pattern (Fig. 1e), it is found that the main diffraction spots correspond to the (440) facet (the secondary diffraction of the (220) facet) and the (311) facet. And in the selected area electron diffraction pattern (Figure S1b), the diffraction spots corresponding to the (311) and (220) facets are clearly observed. Then using the scanning transmission electron microscopy electron energy loss spectroscopy (STEM-EELS) technique, as shown in Fig. 1f, a uniform elemental distribution is evidenced for the catalyst particles.

### H_2_O_2_ generation performance through selective water oxidation

**Fig. 2:**
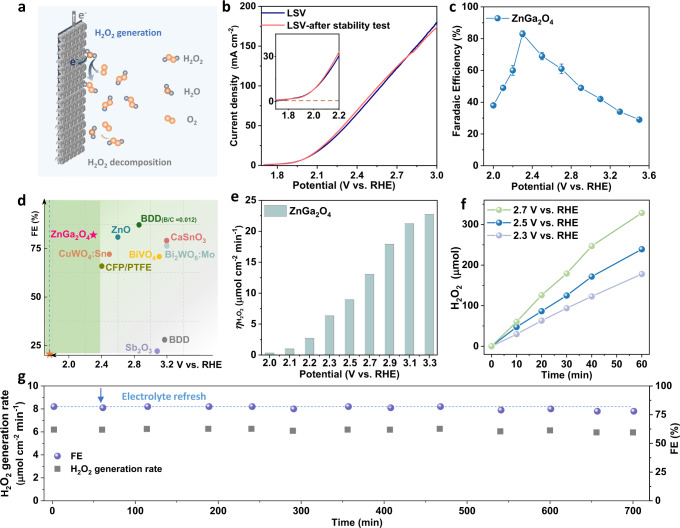
Activity and stability of H_2_O_2_ generation on ZnGa_2_O_4_ anode. **a** Scheme of the electrochemical H_2_O_2_ production on anodes through a water oxidation reaction. **b** LSV curves recorded in 2 M KHCO_3_ before and after a stability test. The scan rate is 5 mV s^−1^. **c** H_2_O_2_ FE of the prepared ZnGa_2_O_4_ anode at different potentials in 2 M KHCO_3_ electrolyte (Error bars represent the standard deviation of three independent measurements). **d** The FE of typically reported anodes including ZnO^25^, boron-doped diamond (BDD)^33^, BDD (B/D ratio = 0.012)^39^ BiVO_4_^38^, CaSnO_3_^24^, Bi_2_WO_6_:Mo^7^, and Sb_2_O_3_^26^ for H_2_O_2_ generation in bicarbonate/carbonate electrolyte for comparison. The orange star represents the thermodynamic potential of 2e-WOR. **e** The H_2_O_2_ generation rate on the ZnGa_2_O_4_ anode at different potentials. **f** The accumulated amount of H_2_O_2_ in 60 min-electrolysis under different applied potentials, (50 mL electrolyte, electrode area = 0.5 cm^2^). **g** The FE and generation rate of H_2_O_2_ on ZnGa_2_O_4_ anode with an applied bias of 2.3 V versus RHE in 700 minutes. The electrolyte was periodically replaced.

The ZnGa_2_O_4_ anode was designed for anodic H_2_O_2_ production (design principle can be found in Method part). As shown in Figure S2, most ZnGa_2_O_4_ particles are in single-particle dispersion on the anode surface rather than in agglomerates. For the electrochemical water oxidation reaction, as shown in Fig. 2a, the generation of H_2_O_2_ is accompanied by the decomposition of H_2_O_2_ on the anode surface (oxidation of H_2_O_2_ to O_2_) or in the bulk solution (Eq. (4)). The H_2_O_2_ generation performance evaluation is based on the accumulated H_2_O_2_ concentration in the anolyte. From the LSV curves in Fig. 2b, the overpotential at 10 mA cm^−2^ of the ZnGa_2_O_4_ anode was about 270 mV, suggesting a good water oxidation activity of the ZnGa_2_O_4_ anode. Next, the FE of H_2_O_2_ was evaluated in a potential range from 2.0 to 3.5 V versus RHE (details can be found in Table S2), and the corresponding current density was shown in Figure S3a. From Fig. 2c, the ZnGa_2_O_4_ anode shows a FE as high as 38% at 2.0 V versus RHE. Significantly, the peak FE reaches 82% at 2.3 V versus RHE (overpotential of 540 mV), representing a high selectivity of ZnGa_2_O_4_ towards H_2_O_2_ formation. As shown in Fig. 2d, for example, maximum FE of 71% at 3.1 V versus RHE on BiVO_4_^38^, 76% at 3.2 V versus RHE on CaSnO_3_^24^, 81% at 3.1 V versus RHE on ZnO^25^, 28% at 3.17 V versus RHE on boron-doped diamond (BDD)^33^, 87% at 2.85 V versus RHE on BDD (B/D ratio = 0.012)^39^, 22% at 3.08 V versus RHE on Sb_2_O_3_^40^, 79% at 3.2 V versus RHE on Bi_2_WO_6_:5%Mo^7^, 8% at 2.9 V versus Ag/AgCl on C,N codoped-TiO_2_ in an electrolyte of pH = 3^35^. In comparison to these reported anodic materials that usually require overpotentials larger than 1000 mV to afford a high selectivity, the developed ZnGa_2_O_4_ anode shows competitive FE at a low overpotential of 540 mV. Meanwhile, as shown in Table S3, the current density of the ZnGa_2_O_4_ anode is comparable to the reported performance at similar potentials. And the H_2_O-to-H_2_O_2_ partial current density (Figure S3b) is higher than that of previous oxide anodes^26,28,38^. At a potential equal to or higher than 2.3 V versus RHE, O_2_ bubbles were observed on the anode surface, which is considered from the O_2_ evolution reaction (OER).

The generation rate is another important parameter in evaluating the performance of catalysts. The H_2_O_2_ generation rate of the ZnGa_2_O_4_ anode at different potentials was measured (Fig. 2e). The generation rate exceeds 10 μmol cm^−2^ min^−1^ at potentials higher than 2.7 V versus RHE. Such a high generation rate can meet the requirement of H_2_O_2_ for many applications, including water disinfection (>1 mM)^41^, and Fenton reaction (>1 mM)^42–44^. In addition, it is found that higher concentrations of KHCO_3_ lead to higher FE and generation rates for H_2_O_2_ production (Figure S4). Then the accumulated amounts of H_2_O_2_ in 60 minutes under different potentials (2.3, 2.5, and 2.7 V versus RHE) were recorded. The product formed on the anode would be desorbed into bulk solution and accumulates to high concentrations over time. As shown in Fig. 2f, the real-time concentration of H_2_O_2_ increases almost linearly in 60 minutes, which indicates that the decomposition rate is not notable compared to the generation rate of H_2_O_2_. Finally, the cycle water oxidation tests were conducted at 2.3 V versus RHE (Figure S5 and Fig. 2g). The electrolyte was periodically replaced to minimize the effect of changes in electrolyte composition. The H_2_O_2_ FE was measured for each test cycle (Fig. 2g), and a FE of ~78% was maintained after 700 min of continuous electrolysis. It also reveals that the generation rate decreased from 6.27 to 5.95 μmol cm^−2^ min^−1^ after 700 minutes of stability test. The LSV curve (Fig. 2b) was also recorded after the stability test and no apparent changes were observed in the comparison. The flower-like morphology of the ZnGa_2_O_4_ catalyst on the anode surface was retained after the stability test (Figure S6). By comparing the high-resolution X-ray photoelectron spectroscopy spectra of Zn 2*p* and Ga 3*d* obtained before and after the stability test, it can be inferred that the valence state of metal ions in this spinel structure is stable after the electrolysis test (Figure S7). The nearly unchanged LSV curves before and after the stability test, the slightly decreased FE after the 700-min electrolysis, and the unchanged morphology indicate the excellent durability of ZnGa_2_O_4_ catalyst under water oxidation conditions.

According to recent progress, anodic H_2_O_2_ production was also achieved in carbonate-based electrolytes^45–47^. Here, the H_2_O_2_ production performance of ZnGa_2_O_4_ anode in 2 M K_2_CO_3_ solution was then investigated (Figure S8). The ZnGa_2_O_4_ can afford a much higher current density in 2 M K_2_CO_3_ (Figure S8a) than in 2 M KHCO_3_ (Fig. 2b). The highest FE reaches 77%, but the corresponding potential is as high as 2.9 V versus RHE (Figure S8b). Figure S8c shows that the highest H_2_O_2_ generation rate of ~69 μmol cm^−2^ min^−1^ was achieved at 3.1 V versus RHE, which is much higher than that in 2 M KHCO_3_ electrolyte (21 μmol cm^−2^ min^−1^ at 3.1 V versus RHE). Next, at 2.9 V versus RHE, H_2_O_2_ concentration reaches 54 mM after 150 minutes (Figure S8d).

### Decomposition of H_2_O_2_ during electrolysis

**Fig. 3:**
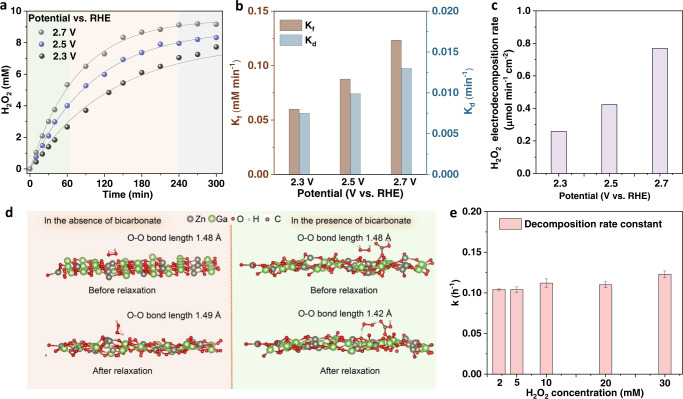
The accumulation and decomposition of H_2_O_2_. **a** The real-time H_2_O_2_ concentration as a function of time at 2.3, 2.5, and 2.7 V versus RHE. (60 mL electrolyte, electrode area = 0.5 cm^2^). The solid lines are the fitting curves. **b** The formation rate constant and decomposition rate constant of H_2_O_2_ on ZnGa_2_O_4_ anode at different potentials. **c** Electrodecomposition rate of H_2_O_2_ on ZnGa_2_O_4_ anode at different potentials in 2 M KHCO_3_. **d** Structure of H_2_O_2_-ZnGa_2_O_4_ before and after relaxation in the absence of bicarbonate (left) and the presence of bicarbonate (right). **e** Self-decomposition rate constants of H_2_O_2_ with different initial concentrations in 2 M KHCO_3_ solution. Error bars represent the standard deviation of three independent measurements.

The real-time concentration of H_2_O_2_ in the anolyte over several hours is essential for realistic applications. Herein, the H_2_O_2_ concentration in 300 minutes was recorded under potentials of 2.3, 2.5, and 2.7 V versus RHE (Fig. 3a), and the corresponding current density can be found in Figure S9. Figure 3a reveals that the H_2_O_2_ concentration curves deviate obviously from the linear growth trend after 60 minutes-electrolysis and then reach a plateau after 240 min-electrolysis. The declining growth rate should be related to the decomposition of high concentrations of H_2_O_2_ in the electrolyte. At constant potential, the formation rate of H_2_O_2_ can be described as zero-order kinetics, and the decomposition of H_2_O_2_ follows the first-order kinetics related to the H_2_O_2_ concentration ([H_2_O_2_])^48–50^. The formation rate (r_f_) equals to *K*_f_ (*K*_f_: formation rate constant); the decomposition rate (*r*_d_) equals *K*_d_[H_2_O_2_], (*K*_d_: decomposition rate constant). Thus, the real-time H_2_O_2_ concentration can be expressed as:

5 $$[{{{{{{\rm{H}}}}}}}_{2}{{{{{{\rm{O}}}}}}}_{2}]=({{{{{{\rm{K}}}}}}}_{{{{{{\rm{f}}}}}}}/{{{{{{\rm{K}}}}}}}_{{{{{{\rm{d}}}}}}})[1-{{\exp }}(-{{{{{{\rm{K}}}}}}}_{{{{{{\rm{d}}}}}}}{{{{{\rm{t}}}}}})].$$

Then according to the fitting results (Fig. 3a), the *K*_f_ under 2.3, 2.5 and 2.7 V versus RHE were calculated to be 0.06, 0.088, and 0.123 mM min^−1^, respectively. The corresponding *K*_d_ under 2.3, 2.5, and 2.7 V versus RHE was calculated to be 0.008, 0.01, and 0.013 min^−1^ (Fig. 3b). These results indicate that the formation and decomposition rate constants (i.e., *K*_f_, *K*_d_) are increased at higher potentials. In consequence, the decomposition rate (*r*_*d*_) increases at more positive potentials.

As shown in Figure S10, the apparent generation rates of H_2_O_2_ in 300 min-electrolysis gradually decrease over time because of H_2_O_2_ decomposition, which can be caused by the anode materials (electrodecomposition and chemical decomposition on anode surface), H_2_O_2_ disproportionation in the electrolyte, and the changes of the anolyte composition^46^ during electrolysis. To evaluate the electrodecomposition of H_2_O_2_ on the ZnGa_2_O_4_ anode, a current integration method (details can be found in the Method) was used^51^. As shown in Figure S11, at 2.3 and 2.5 V versus RHE, there is no significant increase in the current when adding 10 mM H_2_O_2_ to the electrolyte. The corresponding electrodecomposition rates are calculated to be 0.26, 0.42, and 0.77 μmol cm^−2^ min^−1^ at 2.3, 2.5, and 2.7 V versus RHE (in Fig. 3c), suggesting the higher electrodecomposition rates of H_2_O_2_ at more positive potentials. Therefore, the ZnGa_2_O_4_ anode affording high FE at a low potential can help to suppress the electrodecomposition of H_2_O_2_. Meanwhile, it has been reported that catalysts themselves can also induce the chemical decomposition of H_2_O_2_^52^. Figure S12 shows a negligible difference in H_2_O_2_ stability between the control experiments, indicating that ZnGa_2_O_4_ would not decompose the generated H_2_O_2_. The influence of bicarbonate on H_2_O_2_ stability was then also explored, as adsorbed bicarbonate species have been reported to decompose the formed H_2_O_2_^53^. The stability of the peroxy bond of H_2_O_2_ on ZnGa_2_O_4_ surface with and without bicarbonate anions was also examined by theoretical calculations. The bond length of O-O almost maintains its initial value after structure relaxation (Fig. 3d left). This indicates that the peroxy bond is less likely to decompose on the surface of ZnGa_2_O_4_. Similarly, on the surface of ZnGa_2_O_4_ with bicarbonate adsorbed, the bond length of O-O does not increase and the structure of H_2_O_2_ does not change greatly (Fig. 3d right). As a result, two hydrogen bonds are formed with one linked to the bicarbonate and another one linked to the oxygen atom from the ZnGa_2_O_4_ substrate, suggesting the H_2_O_2_ is less likely to decompose on the surface of ZnGa_2_O_4_ with adsorbed bicarbonate.

In addition to the decomposition on the anode, H_2_O_2_ would undergo disproportionation (Eq. (4)) in weak alkaline solutions or with the presence of metal impurities (usually transition-metal ions)^54^. Figure S13 records the remaining ratio of H_2_O_2_ with different initial concentrations ([H_2_O_2_]_0_) as a function of time. More than 60% of H_2_O_2_ self-decomposed after 360 minutes at room temperature when [H_2_O_2_]_0_ is in the range of 2–30 mM, indicating that the disproportionation can cause severe loss of H_2_O_2_. Similarly, a decrease of 19% in H_2_O_2_ concentration (from 1.1 to 0.89 mM) at an open circuit after 150 minutes was reported by Pangotra et al.^46^. The linear fitting curves in Figure S13 indicate that the disproportionation of H_2_O_2_ in 2 M KHCO_3_ solution is a zero-order reaction. Accordingly, the decomposition rate constants are determined to be 0.10 ~ 0.12 h^−1^ (Fig. 3e, details can be found in Figure S13). At last, the changes in conductivity and pH of anolyte were explored. In Figure S14, a decrease in conductivity (~ 3%) and an increase in pH (from 8.31 to 8.68) were recorded at 2.3 V versus RHE during the 300 minutes-electrolysis. It is considered that such slight changes in the electrolyte were not the main factors for the declined H_2_O_2_ generation rate. Therefore, for the anodic H_2_O_2_ production in the absence of a stabilizer, an integrated effect of H_2_O_2_ decomposition (in the bulk solution and at the anode surface) as well as the changes in electrolytes leads to the concentration plateau during continuous electrolysis. For the ZnGa_2_O_4_ anode, the above evidence reveals that disproportionation instead of the electrode composition is mainly responsible for the H_2_O_2_ concentration plateau.

### Proposed reaction pathways

**Fig. 4:**
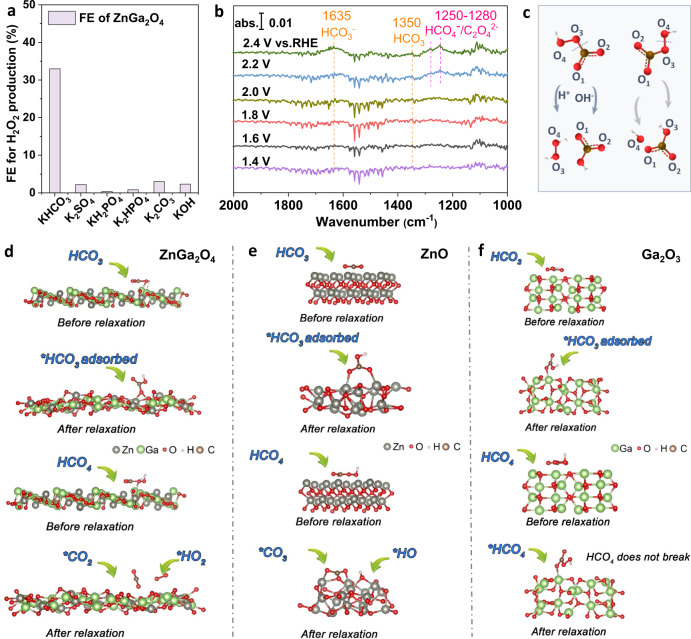
The bicarbonate species-mediated reaction pathway. **a** The H_2_O_2_ FE of ZnGa_2_O_4_ anode in different electrolytes (0.5 M) at a constant current of 8 mA cm^−2^. **b** In situ Fourier transform infrared spectra of ZnGa_2_O_4_ at different potentials in 2 M KHCO_3_. **c** Two possible decomposition ways of HCO_4_ on the catalyst surface. Models of **d** HCO_3_-ZnGa_2_O_4_ and HCO_4_-ZnGa_2_O_4_ at (311) facet, **e** HCO_3_-ZnO and HCO_4_-ZnO, **f** HCO_3_-Ga_2_O_3_ and HCO_4_-Ga_2_O_3_ before and after structure relaxation.

Then the reaction pathway is further investigated. First, the ZnGa_2_O_4_ anode was tested in other electrolytes including K_2_SO_4_, K_2_HPO_4_, KH_2_PO_4,_ K_2_CO_3,_ and KOH to study the effect of electrolyte composition on H_2_O_2_ generation. Considering that the above electrolytes have different oxidation potentials, the FE of H_2_O_2_ in different electrolytes was also tested at a constant current density (8 mA cm^−2^) by the chronopotentiometry method. The results are shown in Fig. 4a and Figure S15, it is found that the FE of H_2_O_2_ in 0.5 M KHCO_3_, 0.5 M K_2_SO_4_, 0.5 M KH_2_PO_4_, 0.5 M K_2_HPO_4_, 0.5 M K_2_CO_3_, and 0.5 M KOH are 32.5%, 2%, 0.3%, 0.8%, 3%, 2.3%, respectively. These observations indicate that HCO_3_^−^ anions play an indispensable role in tailoring the selectivity of water oxidative reaction on the ZnGa_2_O_4_ anode. At potentials above 1.9 V versus RHE, it is accepted that the reaction mainly follows a direct water oxidation pathway (H_2_O/OH^−^ to H_2_O_2_) in K_2_SO_4_, K_2_HPO_4_, and KH_2_PO_4_. However, in the presence of HCO_3_^-^, competing reactions involving the oxidation of HCO_3_^−^ may occur on the anode surface (*E*(HCO_4_^−^/HCO_3_^−^) = 1.8 ± 0.1 V versus NHE)^29^. To gain insight into the evolution of HCO_3_^−^ on the anode, experimental and theoretical investigations were conducted to explore the adsorption properties of these species.

### The ${{HCO}}_{3}^{-}$-mediated water oxidation pathway

The effect of bicarbonate on boosting FE of water oxidation towards H_2_O_2_ has ever been observed by our group^8^ and others^29,55^. However, this proposed reaction pathway needs more solid evidence and in-depth investigation. Here, in situ attenuated total reflectance Fourier transform infrared spectroscopy (ATR-FTIR) technique was used to probe the reaction intermediates. A schematic representation of the electrochemical cell used for ATR-FTIR test can be found in Figure S16. In Fig. 4b, above 1.8 V versus RHE, the FTIR peaks at 1635 cm^−1^ and 1350 cm^−1^ are related to the symmetric C-O stretch of the adsorbed HCO_3_^−^^56^. When the applied potential increased to 2.2 V versus RHE, new FTIR bands were observed, and the intensity of bands become stronger at 2.4 V versus RHE. Specifically, the FTIR bands at 1286 cm^−1^ (valence C=O sym.) and 1265 cm^−1^ (C=O) can be matched to the symmetric C=O stretch of HCO_4_^−^/C_2_O_6_^2-^^57,58^, indicating the formation of percarbonate species on the surface of ZnGa_2_O_4_. Therefore, the in situ spectral identifications prove the indirect reaction pathway for H_2_O_2_ generation via the transformation of bicarbonate to percarbonate species.

Next, models with ${{{{{{\rm{HCO}}}}}}}_{3}^{-}$ or ${{{{{{\rm{HCO}}}}}}}_{4}^{-}$ on the anode surface are constructed to explore the adsorption properties of bicarbonate species. The oxygen atoms of HCO_4_^-^ are labeled for convenient discussion (Fig. 4c). As shown in Fig. 4d, after structure relaxation, the HCO_3_^−^ could be adsorbed stably on the (311) facet of ZnGa_2_O_4_ by forming a bridged adsorption configuration (the O_1_ atom of *HCO_3_ binding with two Ga atoms). The adsorption energy is calculated to be −1.9 eV according to the following equation:

6 $${{{{{{\rm{E}}}}}}}_{{{{{{\rm{ad}}}}}}}={{{{{{\rm{E}}}}}}}_{{{{{{\rm{tot}}}}}}}-{{{{{{\rm{E}}}}}}}_{{{{{{\rm{sub}}}}}}}-{{{{{{\rm{E}}}}}}}_{{{{{{\rm{inter}}}}}}}.$$

Where *E*_ad_ is the adsorption energy, *E*_tot_ is the total energy of the system, *E*_sub_ is the energy of the substrate and *E*_inter_ is the energy of HCO_3_^−^ intermediate. The negative value of *E*_ad_ confirms that the HCO_3_^−^ adsorption on the (311) facet of ZnGa_2_O_4_ is favorable. As revealed by the in situ ATR-FTIR spectra, the adsorbed bicarbonate (*HCO_3_) can be oxidized to percarbonate species (*HCO_4_) on the surface of ZnGa_2_O_4_. Meanwhile, we also used theoretical calculations to study the energy change of the system (Theoretical calculations can be found in Method). In the theoretical calculations, (311), (220) and ($\bar{1}$12) facets were all considered. On (311) facet, the energy of the HCO_4_-ZnGa_2_O_4_ system decreases by 1.82 eV compared to that of HCO_3_-ZnGa_2_O_4_ at 2.3 V versus RHE at 298 K (Fig. 4d). The energy decreases by 0.16 eV on the (220) facet and decreases by 0.39 eV on the ($\bar{1}$12) facet at 2.3 V versus RHE at 298 K (Figure S17, S18, the energy changes are summarized in Table S4). These results indicate that the conversion of *HCO_3_ to *HCO_4_ on (311), (220), and ($\bar{1}$12) facets of ZnGa_2_O_4_ is thermodynamically favorable. It is inferred that the conversion of *HCO_3_ to *HCO_4_ originates from the special dual adsorption sites of *HCO_3_ on ZnGa_2_O_4_.

Then to gain insight into the HCO_3_^−^ mediated pathway on the ZnGa_2_O_4_ anode, the adsorption properties of bicarbonate and percarbonate on ZnO and Ga_2_O_3_ were also investigated for comparison. For the model of ZnO-*HCO_3_, two oxygen atoms (O_1_ and O_2_) of *HCO_3_ adsorb with two Zn atoms (Fig. 4e top) separately, suggesting ZnO is favorable for *HCO_3_ adsorption. In the case of Ga_2_O_3_, the *HCO_3_ adsorbs on the surface of Ga_2_O_3_ by forming a single adsorption site of Ga (Fig. 4f). The formed HCO_4_^-^ species on the anode surface can be desorbed into the bulk electrolyte, followed by a hydrolysis process to give H_2_O_2_^55^. Alternatively, as a highly reactive species, the HCO_4_^-^ may also undergo decomposition on the anode surface. According to DFT results, in the models of HCO_4_-ZnGa_2_O_4_ (Fig. 4d bottom, S17b, and S18b), the *HCO_4_ will decompose into *CO_2_ and *O_2_H by cleaving the -C-O_3_- bond after structure relaxation. Then the *CO_2_ will dissolve into the solution and form HCO_3_^-^ by combing OH^-^ anions, while *O_2_H, containing the -O-O- bond, would transform to H_2_O_2_. In such transformation, the peroxy bond keeps intact, representing one of the advantages of the ZnGaO_4_ anode. However, on the surface of ZnO, the *HCO_4_ would decompose to *CO_3_ and *OH by breaking the –O_3_–O_4_- bond (Fig. 4e). This may be caused by the strong interaction between the O atoms of percarbonates and the Zn sites. This decomposition may also be affected by the initial adsorption states. As shown in Fig. 4e, the O_1_ and O_2_ atoms of the *HCO_3_ form two Zn-O bonds with the ZnO substrate. This is different from the situation on ZnGa_2_O_4_, where two Ga are bonded with only one oxygen atom of _*_HCO_3_. Although both cases are dual-site adsorption, the –C–O_3_- bond of *HCO_4_ is less likely to be cleaved on the ZnO model. Instead, the *HCO_4_ will decompose to *CO_3_ and *OH by breaking the peroxy bond on ZnO. Then three O atoms of the *CO_3_ are bonded with Zn atoms as shown in Fig. 4e. In the case of Ga_2_O_3_, the *HCO_4_ is just stably adsorbed on the anode surface (Fig. 4f), where the O_1_ or O_2_ atom of *HCO_4_ is bonded with a Ga site. Such adsorption configuration avoids the decomposition of *HCO_4_.

### The OH^-^-mediated water oxidation pathway

**Fig. 5:**
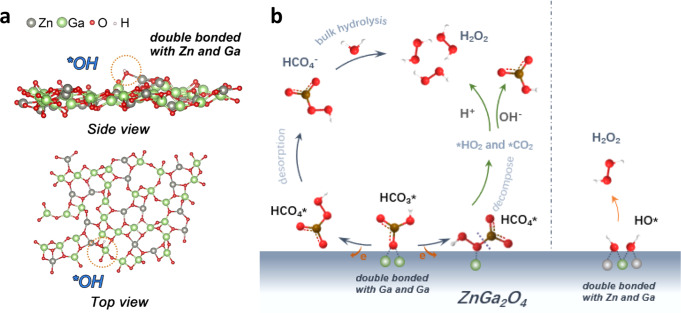
Proposed reaction pathways of water oxidation to H_2_O_2_. **a** Side view and top view of adsorption of *OH on ZnGa_2_O_4_. **b** Scheme illustration of the proposed direct and indirect pathways toward H_2_O_2_ generation on the ZnGa_2_O_4_ surface.

The generation of H_2_O_2_ in electrolytes without bicarbonate (Fig. 4a) indicates the direct water oxidation reaction on ZnGa_2_O_4_ anode. Prior studies have proposed that the adsorption energy of *OH (ΔG_*OH_) on the surface of the catalyst determines the selectivity of WOR^38,59^. Small ΔG_*OH_ often causes the evolution of O_2_ while large ΔG_*OH_ leads to the formation of ·OH radicals. To produce H_2_O_2_, a suitable ΔG_*OH_ should be smaller than 2.4 eV and larger than 1.6 eV. According to DFT results, after relaxation, it is discovered that *OH can adsorb on the surface of ZnGa_2_O_4_ with an energy of 1.63 eV, which is within the optimum range for H_2_O_2_ generation. Interestingly, the O atom in *OH is bonded with Zn and Ga atoms (Fig. 5a). The bond length of Zn-O is 1.97 Å, and the bond length of Ga-O is 2.04 Å. The similar bond length confirms the formation of a dual-site (Zn-Ga) adsorption configuration. As a result, the water oxidation to H_2_O_2_ molecules is reasonably inferred at this Zn-Ga region with a dual-adsorption site. A detailed energy diagram of the reaction steps involved in the OH^-^-mediated water oxidation pathway was shown in Figure S19. In addition, as shown in Figure S20, the distinct advantage of the HCO_3_-mediated pathway in promoting anodic H_2_O_2_ generation was also observed on some other anodes including F-doped tin oxide (FTO), FTO/WO_3_, and Toray TGP-H-060 carbon fiber paper (CFP). For example, the H_2_O_2_ FE of FTO, FTO/WO_3_, and CFP are 5%, 13%, and 9% in 0.5 M KHCO_3_. However, the H_2_O_2_ FE is below 0.5% for all three anodes in 0.5 M KOH.

Overall, on this ZnGa_2_O_4_ anode, H_2_O_2_ can be generated through either the HCO_3_^-^-mediated reaction pathway or the OH^-^-mediated reaction pathway (Fig. 5b). The former plays a critical role in boosting H_2_O_2_ FE. Meanwhile, DFT results indicate that the conversion of *HCO_3_ to *HCO_4_ relies on the dual-adsorption site (Ga-Ga) of ZnGa_2_O_4_. Furthermore, percarbonate species may undergo decomposition on the electrode surface, giving rise to *CO_2_ and*HO_2_ by breaking the –C–O– bond or *CO_3_ and *HO by breaking the -–O–O– bond (Fig. 5b). The latter causes a decrease in FE because the crucial intermediate HCO_4_^-^ was eliminated. The theoretical studies indicate that the high H_2_O_2_ FE of ZnGa_2_O_4_ is related to the conversion of *HCO_3_ to *HCO_4_ at the dual-site, and to the intact peroxides on the surface of ZnGa_2_O_4_. Therefore, the FE is not only determined by the reaction selectivity but also influenced by the stability of the peroxy bond on the anode surface.

Next, the real-time H_2_O_2_ concentration was recorded when applying a potential of 2.7 V versus RHE to the anode (Figure S21). It is found that the H_2_O_2_ concentration increases linearly in the first 60 minutes and then the growth slows down. The H_2_O_2_ concentration reaches a plateau after 200 minutes owing to H_2_O_2_ disproportionation and electrodecomposition. After 300 min-electrolysis, the H_2_O_2_ concentration reaches ~22 mM. It is worth noting that such a concentration has met the requirement of applications such as the Fenton reaction (> 1 mM)^42,60,61^, water disinfection (>1 mM)^41^.

## Discussion

In conclusion, a highly selective ZnGa_2_O_4_ anode was developed for H_2_O_2_ production by oxidizing water, delivering a high FE of 82% at a low overpotential of 540 mV, along with good stability. In this case, it is revealed that the H_2_O_2_ concentration plateau during continuous electrolysis up to 5 h is largely realted to homogeneous decomposition (disproportionation) instead of electro decomposition. Based on our experimental observations and the theoretical calculations, the H_2_O_2_ is formed on ZnGa_2_O_4_ through both the indirect and direct reaction routes. More importantly, the indirect route, mediated by the HCO_3_^-^/HCO_4_^-^, is the critical route for H_2_O_2_ production. ZnGa_2_O_4_ favors the adsorption of HCO_3_^-^ at the dual Ga-Ga sites. The conversion of *HCO_3_ to *HCO_4_ on ZnGa_2_O_4_ is confirmed by the in situ ATR-FTIR spectra and the energy change in DFT results. Significantly, adsorbed *HCO_4_ was found to decompose into *CO_2_ and *O_2_H, and the latter would transform into H_2_O_2_. Additionally, the peroxy bond in H_2_O_2_ and HCO_4_^-^ is stable on the ZnGa_2_O_4_ surface with and without HCO_3_^-^ anions.

As well-known, the anodic H_2_O_2_ generation performance can be affected by the supporting electrolyte^27,45,46,62^. For this ZnGa_2_O_4_ anode, given the high FE in bicarbonate as well as the large current density in carbonate solution for anodic H_2_O_2_ generation, further study can be focused on optimizing the composition of electrolytes further to improve the FE of ZnGa_2_O_4_ for H_2_O_2_ production and to elucidate the reaction mechanism behind it.

## Methods

### Preparation of catalyst and electrodes

The ZnGa_2_O_4_ anode was designed for anodic H_2_O_2_ formation because of the following reasons. Firstly, it is important to avoid the decomposition effect of the catalyst itself on H_2_O_2_. Thus, some metal cations cannot be chosen. For example, catalysts that can decompose H_2_O_2_ into O_2_^29,52^ (MnO_2_ and CoO) or catalysts containing Fe or Mn elements that can activate H_2_O_2_ into hydroxide radicals^63^ should be excluded. Secondly, to improve the selectivity of H_2_O_2_, competitive reactions such as oxygen evolution and the formation of hydroxyl radicals should be suppressed on the anode surface. The strength of oxygen chemisorption on transition metal (TM) ions should be taken into consideration when designing the catalysts for electrochemical water oxidation. Numerous efforts have revealed that the oxygen chemisorption strength on a metal surface is related to its electronic structure, i.e. the position of the metal’s d-band center relative to its Fermi level^64–67^. Those TM atoms such as Co, Ni, Fe, Mn featuring suitable chemisorption of oxygen on the catalyst surface were excluded because *OH can be easily dissociated into *O and finish the 4e-water oxidation pathway. Thirdly, according to the previous researches, the most reported anode materials for H_2_O_2_ production are those TM oxides containing cationic ions with closed electronic shell configurations, such as W^6+^ (5d^0^), Bi^3+^ (6s^2^), Zn^2+^ (3d^10^), Sn^4+^ (4d^10^). It is possible that the dominance of this configuration is related to the inert oxygen evolution activity of these metal oxides. Therefore, we designed ZnGa_2_O_4_ anode in which Zn and Ga have closed electronic shell configurations to investigate the anodic H_2_O_2_ generation performance. Besides, the good chemical stability of ZnGa_2_O_4_ in oxidative condition is also beneficial to 2e-WOR, which has been demonstrated in previous studies^37,68–70^.

The ZnGa_2_O_4_ microsphere was prepared according to a previous report with minor modifications^37^. Gallium nitrate hydrate and zinc acetate were dissolved in pure water (10 mL) with a molar ratio of 2:1. To obtain the flower-like structure consisting of nanosheets, the concentration of zinc precursor was optimized to 25 mM. Then 5 mL of ethylenediamine was added to the above solution and stirred at room temperature for 20 min. Next, the mixture was transferred to a 25 mL autoclave and heated at 200 °C for 16 h. After cooling down to room temperature, the sample was obtained by centrifugation and washed with deionized water and ethanol several times in sequence. Then the sample was dried at 60 °C overnight. After cleaning with ethanol three times, the carbon fiber cloth (Taiwan Tanneng, WOS1009) was dried at room temperature. Next, 300 mg ZnGa_2_O_4_ catalyst and 30 mg polytetrafluoroethylene were dispersed in 10 mL ethanol, followed by a sonication treatment for 20 min to form a highly dispersed mixture. Then the cleaned CC was placed on a hot plate (150 °C), and the ZnGa_2_O_4_ catalyst mixture was painted onto the CC by brushing, followed by a heat treatment at 350 °C for 30 min in Argon flow. The loading amount of ZnGa_2_O_4_ was about 20 mg cm^−2^.

### Characterizations

Phase identification was conducted on a Rigaku SmartLab X-ray diffractometer with Cu Kα irradiation (λ = 1.5406 Å) operation at 40 kV and 40 mA. X-Ray photoelectron spectroscopy (XPS) characterizations of samples before and after electrolysis or Fenton reaction were obtained on a Thermo ESCALAB 250Xi spectrometer equipped with an anode of Al Kα radiation (1486.6 eV) X-ray sources. The obtained XPS data were calibrated by the C1*s* position (284.8 eV) before the data process. For the quantification of the formed H_2_O_2_ from anode under different biases, the concentration of H_2_O_2_ was quantified based on the spectrophotometric determination of I_3_^−^^71^. Firstly, solutions A and B were prepared for the quantification of H_2_O_2_ by I_3_^−^ method. To prepare solution A, 33 g KI, 1.4 g KOH, and 0.1 g (NH_4_)_6_Mo_7_O_24_·4H_2_O were dissolved in 500 mL distilled water. 10 g potassium hydrogen phthalate was dissolved in 500 mL distilled water as solution B. Solution A should be kept in the dark to inhibit the oxidation of I^−^. The pH of the sample solution was adjusted to 7 before the test. Then the sample solution, solutions A and B were mixed in equal volumes. After 5 min, the UV absorption spectrum of the mixture solution was recorded on Cary Series UV-Vis Spectrometer (Agilent Technologies). The accumulated concentration of H_2_O_2_ in the anolyte was quantified, based on which the faradaic efficiency and production rate are calculated. Scanning electron microscopy (SEM) characterizations were performed on an FEI Quanta 400 microscope. The transmission electron microscopy (TEM) and high-resolution TEM were conducted on a Philips Tecnai F20 instrument and a CM-120 microscope (Philips, 120 kV).

### ATR-FTIR measurement

For the attenuated total reflectance Fourier transform infrared spectroscopy (ATR-FTIR) test, a single Si crystal (diameter: 20 mm) was used as an ATR crystal. A high-sensitivity mercury calcium telluride (MCT) detector and optical path accessories with high luminous flux were required to detect the adsorption vibration signals. The spectral signal was collected by a Fourier transform infrared spectroscopy (FTIR, Thermo Scientific Nicolet iS50). A rough Au film with good conductivity was deposited onto the ATR crystal to enhance the infrared absorption by means of the local electric field of the surface plasmon. In the three-electrode system, Pt wire was used as the counter electrode, and catalyst film on the Si crystal (coated with Au film) acts as the working electrode connected to the external circuit through a copper foil. A schematic representation of the in situ electrochemical cell for the ATR-FTIR test is shown in Figure S16. The data recorded at open circuit potential was used as the background. All the spectra were recorded in the range of 4000 ~900 cm^−1^ with a resolution of 4 cm^–1^ and averaged with 32 scans.

### Electrochemical measurements

Electrochemical experiments in a three-electrode set-up were performed on a CHI 760E electrochemical station (CH Instruments, Inc., Shanghai). Potentials set against Ag/AgCl were converted to the RHE scale according to E_RHE_ = E_Ag/AgCl_ + 0.197 + 0.059 × pH, and the pH values of electrolytes value were tested by a pH meter (FE28, Mettler Toledo, Switzerland). The conductivity of the electrolyte was measured with a conductivity meter (DDS-307A, Shanghai INESAScientific Instrument Co., Ltd., China). The water oxidation performance of anodes was evaluated in a two-compartment cell divided by a Nafion 117 membrane (DuPond Co.). A Pt foil and a Ag/AgCl electrode (in a Luggin capillary) were used as the counter, and reference electrode, respectively. The electrochemical tests were performed without iR-compensation. Both of the compartments of the H-cell were full with 2 M KHCO_3_ solution, and the electrolyte was stirred at a rate of 600 r.p.m. during the electrochemical test. After passing a particular amount of coulombs (depending on the applied potentials), 0.5 mL anolyte was taken out and the H_2_O_2_ concentration in it was quantified by the I_3_^-^ method. For the cyclic electrochemical tests, the electrolyte was periodically replaced.

### The overpotential is calculated as follows:

7 $${{{{{\rm{Overpotential}}}}}}={E}_{{applied}}-{E}_{{{{{{\rm{thermodynamic}}}}\,{{{\rm{of}}}}}}2{{{{{\rm{e}}}}}}-{{{{{\rm{WOR}}}}}}}={E}_{{applied}}{{{{{\rm{versus}}}}}}\, {{{{{\rm{RHE}}}}}}-1.76{{{{{\rm{V}}}}}}\,{{{{{\rm{versus}}}}}}\, {{{{{\rm{RHE}}}}}}.$$

*E*_*applied*_ is the potential applied to the anode during tests.

### Evaluation on the electrodecomposition of H_2_O_2_ on the ZnGa_2_O_4_ anode

The standardized method for measuring the electrodecomposition (ED) of H_2_O_2_ on anodes during anodic H_2_O_2_ production has yet to be well established. Here, a current integration method, i.e. to compare the faradaic charge with and without H_2_O_2_ (known concentration) at the same applied potential, was used to evaluate the electrodecomposition of H_2_O_2_ on ZnGa_2_O_4_ anode^51^. Specifically, chronoamperometric courses of ZnGa_2_O_4_ anode at potentials (2.3, 2.5, 2.7 V vs. RHE) in 2 M KHCO_3_ solution with and without H_2_O_2_ (10 mM) were recorded (Figure S10). Then the total faradaic charge passed in solution with and without H_2_O_2_ addition was compared, and the difference between the two tests is assumed to be the charge corresponding to the oxidation of H_2_O_2_. Taking the analysis at 2.3 V vs. RHE as an example, the calculation details are shown below.

8 $${\int }_{0s}^{300s}{I}_{{KHCO}3\,{without}\,H2O2}\,{tdt}={Q}_{{KHCO}3\,{without}\,H2O2}$$

9 $${\int }_{0s}^{300s}{I}_{{KHCO}3\,{with}\,H2O2}{tdt}={Q}_{{KHCO}3\,{with}\,H2O2}$$

10 $${{FE}}_{{ED},{current}\,{intergration}}=\frac{{Q}_{{KHCO}3\,{with}\,H2O2}\,-\,{Q}_{{KHCO}3\,{without}\,H2O2}}{{Q}_{{KHCO}3\,{with}\,H2O2}}$$

$$=\frac{3.732\,C-3.578\,C}{3.732\,C}=0.041$$

11 $${{H}_{2}{O}_{2}}_{{{{{{\rm{Electrodecomposition}}}}}}}	={{H}_{2}{O}_{2}}_{{{{{{\rm{Accumulated}}}}}}} \times {{FE}_{{ED},{current}\,{intergration}}} \\ 	=6.27\,\mu {mol}\;{\min }^{-1}{{cm}}^{-2} \times 0.041 \\ 	=0.26\,\mu {mol}\,{\min }^{-1}{{cm}}^{-2}$$

${{H}_{2}{O}_{2}}_{{{{{{\rm{Accumulated}}}}}}}$ means the measured H_2_O_2_ generation rate (6.27 μmol cm^−2^ min^−1^ at 2.3 V vs. RHE in Fig. 2e). ${{FE}}_{{ED},{current\; intergration}}$ at 2.5 and 2.7 V vs. RHE are 0.047 and 0.059. The electro decomposition rate of H_2_O_2_ at 2.5 and 2.7 V vs. RHE in 2 M KHCO_3_ are calculated to be 0.42 and 0.77 μmol cm^-2^ min^-1^.

It should be noted that this method provides an overestimation of the role of H_2_O_2_ electro decomposition because the concentration of H_2_O_2_ added during the tests (10 mM) is higher than the concentration plateau observed in Fig. 3a. Such approximate evaluation of the electro decomposition of H_2_O_2_ can help to identify the main factors causing the decomposition of H_2_O_2_.

### Theoretical calculations

The mechanism for H_2_O_2_ generation is studied by using VASP computational package^7^. Projector-augmented-wave method with the Perdew–Burke–Ernzerhof GGA functional was used^72^. The electronic convergence limit was set to be 1 × 10^−5^ eV^73^. Optimization of atomic coordinates was considered to be converged when Hellmann–Feynman force was smaller than 1× 10^−2^ eV Å^−1^. The established slab of ZnGa_2_O_4_ is (311) facets with Zn and Ga atoms termination. Besides, the established slab of ZnO is (200) facets with Zn atoms termination. The established Ga_2_O_3_ slab is (020) facets with Ga atoms termination. Intermediate including *OH, *HCO_3_ and *HCO_4_ is separately placed on the top of the slab surface. The vacuum region is about 10 Å in height.

The surface termination for the DFT studies was determined based on the XRD pattern, the polycrystalline diffraction patterns, and the HRTEM images. We first choose the (311) facet because it is the strongest diffraction peak in the XRD pattern of the ZnGa_2_O_4_ catalyst, which indicates that (311) facets should widely exist in the crystals. This approach has been used in previous reports on electrocatalysts with a cubic spinel structure, where the theoretical calculations are based on the observed main peaks in the XRD pattern^74–76^. For example, a previous report (Nat. Catal. 2022, 5, 109-118) studied the Co_2_MnO_4_ (space group *Fd*$\bar{3}$*m, a* = *8.0866* *Å*), the DFT calculation was conducted based on the (311) surface (the dominant peak in the PXRD)^75^. Since (311) and (220) facets are also observed in the HRTEM images (Fig. 1d and S1c, d), it can be concluded that (220) and (311) facets widely exist in the prepared ZnGa_2_O_4_ catalyst. It is necessary to investigate the adsorption of reaction intermediates on both (311) and (220) facets. Finally, in the selected area electron diffraction pattern (Figure S1b), considering that the zone axis perpendicular to (311) and (220) facets is [$\bar{1}$12], ($\bar{1}$12) facet is also considered in our calculations.

Next, the conversion of *HCO_3_ to *HCO_4_ on these three facets was investigated. Here, by using the computational hydrogen electrode method to identify the conversion of bicarbonate to percarbonate is thermodynamically favorable or not. The influence of applied potential is considered via computational hydrogen electrode (CHE) method^77,78^. In this method, at standard condition, chemical potential of proton and electron is equals to that of a half of hydrogen gas (μ(H^+^ + e^-^) = 1/2 μ(H_2_)). The potential of the electrons is eU, where e is the elementary charge and U is the potential applied to the electrode.

The catalytic process on the surface is investigated by calculating the energy of intermediates during the process. The transfer of *HCO_3_ to *HCO_4_ should involve the following step:

12 $$\ast {{{{{{\rm{HCO}}}}}}}_{3}^{-}+\ast {{{{{{\rm{OH}}}}}}}^{-}=\ast {{{{{{\rm{HCO}}}}}}}_{4}^{-}+{{{{{{\rm{H}}}}}}}^{+}+2{{{{{\rm{e}}}}}}.$$

Based on the above step, the computational change of energy of the intermediates adsorbed on the surface can be calculated according to the following method:

13 $$\triangle {{{{{\rm{E}}}}}}={{{{{{\rm{E}}}}}}}_{*{{{{{\rm{HCO}}}}}}4}-{{{{{{\rm{E}}}}}}}_{*{{{{{\rm{HCO}}}}}}3}+0.5{{{{{{\rm{E}}}}}}}_{{{{{{\rm{H}}}}}}2}-{{{{{{\rm{E}}}}}}}_{{{{{{\rm{OH}}}}}}}-2{{{{{\rm{eU}}}}}}.$$

Where E_*HCO4_, E_*HCO3_, E_H2_, and E_OH_ is the adsorption energy of HCO_3_, HCO_4_, H_2_, and OH, and U is the applied potential.

The Gibbs free energy changes of intermediates were calculated with zero-point energy, and entropy correction using the equation bellows:

14 $${{{{{\rm{At\; T}}}}}}=0{{{{{\rm{K}}}}}},\,\triangle {{{{{\rm{G}}}}}}=\triangle {{{{{\rm{E}}}}}}+\triangle {{{{{\rm{ZPE}}}}}}-{{{{{\rm{T}}}}}}\triangle {{{{{\rm{S}}}}}}.$$

Where, ZPE, T, and S correspond to zero-point energy, temperature, and entropy, respectively.

In the case of the (311) facet, the E_*HCO4−_E_*HCO3_ is calculated to be −1.00 eV, E_H_ is −3.39 eV, E_OH_ is −7.34 eV, U is 2.3 V. Therefore, ∆E is calculated to be −1.65 eV (E_*HCO4−_E_*HCO3_ + E_H_ - E_OH_ - 2eU).

The ZPE for HCO_3_ is 0.66 eV; ZPE for HCO_4_ is 0.80 eV; ZPE for H is 0.16 eV; ZPE for OH is 0.33 eV; ∆ZPE is −0.03 eV (0.80 eV–0.66 eV + 0.16 eV–0.33 eV = 0.03 eV). At 298 K, TS for HCO_3_ is 0.05 eV; TS for HCO_4_ is 0.14 eV; TS for H is 0.13 eV; TS for OH is 0.07 eV; therefore, T∆S is calculated to be 0.148 eV (0.14 eV–0.05 eV + 0.13 eV–0.07 eV = 0.15 eV). Therefore, ∆ZPE - T∆S is −0.18 eV.

When the temperature effect of enthalpy is considered, a correction of ΔH should be added^79,80^:

15 $$\Delta {{{{{\rm{G}}}}}}=\Delta {{{{{{\rm{E}}}}}}}_{{{{{{\rm{DFT}}}}}}}+\Delta {{{{{\rm{H}}}}}}+\Delta {{{{{\rm{ZPE}}}}}}-{{{{{\rm{T}}}}}}\Delta {{{{{\rm{S}}}}}}.$$

16 $${{{{{\rm{Our}}}}}}\, {{{{{\rm{experiments}}}}}}\, {{{{{\rm{were}}}}}}\, {{{{{\rm{conducted}}}}}}\, {{{{{\rm{at}}}}}} \, 298{{{{{\rm{K}}}}}}.{{{{{\rm{Therefore}}}}}},\,\Delta {{{{{\rm{H}}}}}}={{{{{\rm{H}}}}}}(298{{{{{\rm{K}}}}}})-{{{{{\rm{H}}}}}}\left(0{{{{{\rm{K}}}}}}\right).$$

$${{{{{\rm{H}}}}}}(298{{{{{\rm{K}}}}}})-{{{{{\rm{H}}}}}}(0{{{{{\rm{K}}}}}})=	{{{{{{\rm{H}}}}}}}_{ {\ast} {{{{{\rm{HCO}}}}}}{4}}(298{{{{{\rm{K}}}}}})-{{{{{{\rm{H}}}}}}}_{{{{{{\rm{HCO}}}}}}3}(298{{{{{\rm{K}}}}}})+0.5{{{{{{\rm{H}}}}}}}_{{{{{{\rm{H}}}}}}2}(298{{{{{\rm{K}}}}}})-{{{{{{\rm{H}}}}}}}_{{{{{{\rm{OH}}}}}}}(298{{{{{\rm{K}}}}}}) \\ 	 -({{{{{{\rm{H}}}}}}}_{*{{{{{\rm{HCO}}}}}}4}(0{{{{{\rm{K}}}}}})-{{{{{{\rm{H}}}}}}}_{*{{{{{\rm{HCO}}}}}}3}(0{{{{{\rm{K}}}}}})+0.5{{{{{{\rm{H}}}}}}}_{{{{{{\rm{H}}}}}}2}(0{{{{{\rm{K}}}}}})-{{{{{{\rm{H}}}}}}}_{{{{{{\rm{OH}}}}}}}(0{{{{{\rm{K}}}}}}))$$

$$=	({{{{{{\rm{H}}}}}}}_{*{{{{{\rm{HCO}}}}}}4}(298{{{{{\rm{K}}}}}})-{{{{{{\rm{H}}}}}}}_{*{{{{{\rm{HCO}}}}}}4}(0{{{{{\rm{K}}}}}}))-({{{{{{\rm{H}}}}}}}_{*{{{{{\rm{HCO}}}}}}3}(298{{{{{\rm{K}}}}}})-{{{{{{\rm{H}}}}}}}_{*{{{{{\rm{HCO}}}}}}3}(0{{{{{\rm{K}}}}}})) \\ 	+0.5({{{{{{\rm{H}}}}}}}_{{{{{{\rm{H}}}}}}2}(298{{{{{\rm{K}}}}}})-{{{{{{\rm{H}}}}}}}_{{{{{{\rm{H}}}}}}2}(0{{{{{\rm{K}}}}}}))-({{{{{{\rm{H}}}}}}}_{{{{{{\rm{OH}}}}}}}(298{{{{{\rm{K}}}}}})-{{{{{{\rm{H}}}}}}}_{{{{{{\rm{OH}}}}}}}(0{{{{{\rm{K}}}}}}))$$

$$={{{{{{\rm{C}}}}}}}_{*{{{{{\rm{HCO}}}}}}4}\left(298{{{{{\rm{K}}}}}}{{{{{\rm{{-}}}}}}}0{{{{{\rm{K}}}}}}\right)-{{{{{{\rm{C}}}}}}}_{*{{{{{\rm{HCO}}}}}}3}\left(298{{{{{\rm{K}}}}}}{{{{{\rm{{-}}}}}}}0{{{{{\rm{K}}}}}}\right)+0.5{{{{{{\rm{C}}}}}}}_{*{{{{{\rm{H}}}}}}2}(298{{{{{\rm{K}}}}}}{{{{{\rm{{-}}}}}}}0{{{{{\rm{K}}}}}})-{{{{{{\rm{C}}}}}}}_{{{{{{\rm{OH}}}}}}}(298{{{{{\rm{K}}}}}}-0{{{{{\rm{K}}}}}})$$

Where C_*HCO4_, C_*HCO3_, C_*H2_, and C_OH_ is the heat capacity with the unit of J K^−1^ mol^−1^. C_*HCO4_, C_*HCO3_, C_*H2_, and C_OH_ is 39.6, 30.2, 16.6, and 15.9 J K^-1^ mol^-1^, respectively, which are obtained by using the phonopy in VASP. The unit of J K^-1^ mol^-1^ is converted to eV by dividing 1.6E-19 and NA (6.02E23 mol^-1^). Finally, the correction of H(298 K) - H(0 K) is calculated to be 0.01 eV.

Therefore, at 298 K, the ∆G is calculated to be −1.82 eV (−1.65 eV − 0.18 eV + 0.01 eV) at the potential of 2.3 V versus RHE at 298 K. This negative value suggests that the transformation of *HCO_3_ to *HCO_4_ on the (311) facet is favorable at 2.3 V versus RHE at 298 K.

In the case of the (220) facet, the E_*HCO4−_E_*HCO3_ is 0.66 eV; E_H_ is −3.39 eV; E_OH_ is −7.34 eV; U is 2.3 V. Therefore, ∆E is calculated to be 0.01 eV (E_*HCO4−_ E_*HCO3_ + E_H_–E_OH_- 2eU). The ZPE for HCO_3_ is 0.66 eV; ZPE for HCO_4_ is 0.80 eV; ZPE for H is 0.16 eV; ZPE for OH is 0.33 eV; ∆ZPE is −0.03 eV (0.80 eV- 0.66 eV + 0.16 eV–0.33 eV = 0.03 eV). At 298 K, TS for HCO_3_ is 0.05 eV; TS for HCO_4_ is 0.14 eV; TS for H is 0.13 eV; TS for OH is 0.07 eV; therefore, T∆S is 0.15 eV (0.14 eV–0.05 eV + 0.13 eV - 0.07 eV = 0.15 eV). Therefore, ∆ZPE–T∆S is −0.18 eV.

Therefore, ∆G is calculated to be −0.16 eV at the potential of 2.3 V versus RHE at 298 K. This negative value suggests that at 2.3 V versus RHE the transformation of *HCO_3_ to *HCO_4_ on (220) facet is favorable.

### Reporting summary

Further information on research design is available in the Nature Portfolio Reporting Summary linked to this article.

## Supplementary information

Supplementary Information

Reporting Summary

## Data Availability

The data that support the findings of this study are available within the article and its Supplementary information files. All other relevant data supporting the finding of this study are available from the corresponding authors upon reasonable request. Source data are provided with this paper.

## Source data

Source Data

## References

[1] Tanev PT, Chibwe M, Pinnavaia TJ (1994). Titaniumcontaining mesoporous molecular sieves for catalytic oxidation of aromatic compounds. Nature.

[2] Lane BS, Burgess K (2003). Metal-catalyzed epoxidations of alkenes with hydrogen peroxide. Chem. Rev..

[3] McDonnell-Worth CJ, MacFarlane DR (2018). Progress towards Direct Hydrogen Peroxide Fuel Cells (DHPFCs) as an Energy Storage Concept. Aust. J. Chem..

[4] Ma J, Choudhury NA, Sahai Y (2010). A comprehensive review of direct borohydride fuel cells. Renew. Sustain. Energy Rev..

[5] Fan Z, Kwon Y-H, Yang X, Xu W, Wu Z (2017). In-situ production of hydrogen peroxide as oxidant for direct urea fuel cell. Energy Procedia.

[6] Kosaka K (2001). Evaluation of the treatment performance of a multistage ozone/hydrogen peroxide process by decomposition by- products. Water Res..

[7] Li L, Hu Z, Yu JC (2020). On-demand synthesis of H_2_O_2_ by water oxidation for sustainable resource production and organic pollutant degradation. Angew. Chem. Int. Ed. Engl..

[8] Li L, Xiao K, Wong PK, Hu Z, Yu JC (2022). Hydrogen peroxide production from water oxidation on a CuWO_4_ anode in oxygen-deficient conditions for water decontamination. ACS Appl. Mater. Interfaces.

[9] Campos-Martin JM, Blanco-Brieva G, Fierro JL (2006). Hydrogen peroxide synthesis: an outlook beyond the anthraquinone process. Angew. Chem. Int. Ed. Engl..

[10] Ciriminna R (2016). Hydrogen peroxide: a key chemical for today’s sustainable development. Chemsuschem.

[11] Siahrostami S (2013). Enabling direct H_2_O_2_ production through rational electrocatalyst design. Nat. Mater..

[12] Yang S (2018). Toward the decentralized electrochemical production of H_2_O_2_: A focus on the catalysis. ACS Catal..

[13] Jung E, Shin H, Hooch Antink W, Sung Y-E, Hyeon T (2020). Recent advances on electrochemical oxygen reduction to H_2_O_2_: Catalyst and cell design. ACS Energy Lett..

[14] Shi X, Back S, Gill TM, Siahrostami S, Zheng X (2020). Electrochemical synthesis of H_2_O_2_ by two-electron water oxidation reaction. Chem.

[15] Samanta C (2008). Direct synthesis of hydrogen peroxide from hydrogen and oxygen: An overview of recent developments in the process. Appl. Catal., A.

[16] Li F, Shao Q, Hu M, Chen Y, Huang X (2018). Hollow Pd−Sn nanocrystals for efficient direct H_2_O_2_ synthesis: The critical role of Sn on structure evolution and catalytic performance. ACS Catal..

[17] Wilson NM, Flaherty DW (2016). Mechanism for the direct synthesis of H_2_O_2_ on Pd clusters: Heterolytic reaction pathways at the liquid-solid interface. J. Am. Chem. Soc..

[18] Melchionna M, Fornasiero P, Prato M (2019). The rise of hydrogen peroxide as the main product by metal-free catalysis in oxygen reductions. Adv. Mater..

[19] Zhang J, Zhang H, Cheng MJ, Lu Q (2020). Tailoring the electrochemical production of H_2_O_2_: Strategies for the rational design of high-performance electrocatalysts. Small.

[20] Li L (2020). Tailoring selectivity of electrochemical hydrogen peroxide generation by tunable pyrrolic‐nitrogen‐carbon. Adv. Energy Mater..

[21] Zhang Q (2020). Highly efficient electrosynthesis of hydrogen peroxide on a superhydrophobic three-phase interface by natural air diffusion. Nat. Commun..

[22] Pérez JF (2017). Improving the efficiency of carbon cloth for the electrogeneration of H_2_O_2_: Role of polytetrafluoroethylene and carbon black loading. Ind. Eng. Chem. Res..

[23] Cao P (2021). Durable and selective electrochemical H_2_O_2_ synthesis under a large current enabled by the cathode with highly hydrophobic three-phase architecture. ACS Catal..

[24] Park SY (2018). CaSnO_3_: An electrocatalyst for two-electron water oxidation reaction to form H_2_O_2_. ACS Energy Lett..

[25] Kelly S (2019). ZnO as an active and selective catalyst for electrochemical water oxidation to hydrogen peroxide. ACS Catal..

[26] Miyase Y, Miseki Y, Gunji T, Sayama K (2020). Efficient H_2_O_2_ production via H_2_O oxidation on an anode modified with sb‐containing mixed metal oxides. ChemElectroChem.

[27] Xia C (2020). Confined local oxygen gas promotes electrochemical water oxidation to hydrogen peroxide. Nat. Catal..

[28] Li L (2022). Direct hydrogen peroxide synthesis on a Sn-doped CuWO_4_/Sn anode and an air-breathing cathode. Chem. Mater..

[29] Fuku K, Miyase Y, Miseki Y, Gunji T, Sayama K (2016). Enhanced oxidative hydrogen peroxide production on conducting glass anodes modified with metal oxides. Chemistryselect.

[30] Gill, T. M., Vallez, L. & Zheng, X. The role of bicarbonate-based electrolytes in H_2_O_2_ production through two-electron water oxidation. *ACS Energy Lett*., 2854–2862, (2021).

[31] Mavrikis S, Perry SC, Leung PK, Wang L, Ponce de León C (2021). Recent advances in electrochemical water oxidation to produce hydrogen peroxide: a mechanistic perspective. ACS Sustain. Chem. Eng..

[32] Kuttassery F, Sebastian A, Mathew S, Tachibana H, Inoue H (2019). Promotive effect of bicarbonate ion on two-electron water oxidation to form H_2_O_2_ catalyzed by aluminum porphyrins. ChemSusChem.

[33] Mavrikis S, Göltz M, Rosiwal S, Wang L, Ponce de León C (2020). Boron-doped diamond electrocatalyst for enhanced anodic H_2_O_2_ production. ACS Appl. Energy Mater..

[34] Zhang C (2021). High yield electrosynthesis of hydrogen peroxide from water using electrospun CaSnO_3_@Carbon fiber membrane catalysts with abundant oxygen vacancy. Adv. Func. Mater..

[35] Xue SG (2020). Selective electrocatalytic water oxidation to produce H_2_O_2_ using a C,N codoped TiO_2_ electrode in an acidic electrolyte. ACS Appl. Mater. Interfaces.

[36] Hall SB, Khudaisha EA, Hartb AL (1998). Electrochemical oxidation of hydrogen peroxide at platinum electrodes. Part 1. An adsorption-controlled mechanism. Electrochim. Acta.

[37] Liu Q (2014). Single-crystalline, ultrathin ZnGa_2_O_4_ nanosheet scaffolds to promote photocatalytic activity in CO_2_ reduction into methane. ACS Appl. Mater. Interfaces.

[38] Shi XJ (2017). Understanding activity trends in electrochemical water oxidation to form hydrogen peroxide. Nat. Commun..

[39] Mavrikis S (2021). Effective hydrogen peroxide production from electrochemical water oxidation. ACS Energy Lett..

[40] Wang Y, Lian X, Zhou Y, Guo W, He H (2021). Synthesis and characterization of Sb_2_O_3_: a stable electrocatalyst for efficient H_2_O_2_ production and accumulation and effective degradation of dyes. New J. Chem..

[41] Imlay JA, Linn S (1986). Bimodal pattern of killing of DNA-repair-defective or anoxically grown *Escherichia coli* by hydrogen peroxide. J. Bacteriol..

[42] Li H (2017). Oxygen vacancy associated surface fenton chemistry: surface structure dependent hydroxyl radicals generation and substrate-dependent reactivity. Environ. Sci Technol..

[43] Zeng H (2020). pH-independent production of hydroxyl radical from atomic H*-mediated electrocatalytic H_2_O_2_ reduction: a green fenton process without byproducts. Environ. Sci. Technol..

[44] Hou X (2017). Hydroxylamine promoted goethite surface fenton degradation of organic pollutants. Environ. Sci. Technol..

[45] Mavrikis S, Goltz M, Rosiwal S, Wang L, Ponce de Leon C (2022). Carbonate-induced electrosynthesis of hydrogen peroxide via two-electron water oxidation. ChemSusChem.

[46] Pangotra, D. et al. Anodic production of hydrogen peroxide using commercial carbon materials. *Appl. Catal. B* **303**, 120848 (2022).

[47] Fuku K, Sayama K (2016). Efficient oxidative hydrogen peroxide production and accumulation in photoelectrochemical water splitting using a tungsten trioxide/bismuth vanadate photoanode. Chem. Commun..

[48] Kormann C, Bahnemann DW, Hoffmann MR (1988). Photocatalytic production of H_2_O_2_ and organic peroxides in aqueous suspensions of TiO_2_, ZnO, and desert sand. Environ. Sel. Technol..

[49] Hoffman AJ, Carraway ER, Hoffmann MR (1994). Photocatalytic production of H_2_0_2_ and organic peroxides on quantum-sized semiconductor colloids. Environ. Sci. Technol..

[50] Moon G-h, Kim W, Bokare AD, Sung N-e, Choi W (2014). Solar production of H_2_O_2_ on reduced graphene oxide–TiO_2_ hybrid photocatalysts consisting of earth-abundant elements only. Energy Environ. Sci..

[51] Gill TM, Vallez L, Zheng X (2021). The role of bicarbonate-based electrolytes in H_2_O_2_ production through two-electron water oxidation. ACS Energy Lett..

[52] Izgorodin A, Izgorodina E, MacFarlane DR (2012). Low overpotential water oxidation to hydrogen peroxide on a MnO_x_ catalyst. Energy Environ. Sci..

[53] Nadar A (2020). Evaluating the reactivity of BiVO_4_ surfaces for efficient electrocatalytic H_2_O_2_ production: a combined experimental and computational study. J. Phys. Chem. C.

[54] Lee WT, Xu S, Dickie DA, Smith JM (2013). A robust Mn catalyst for H_2_O_2_ disproportionation in aqueous solution. Eur. J. Inorg. Chem..

[55] Richardson DE, Yao H, Frank KM, Bennett DA (2000). Equilibria, kinetics, and mechanism in the bicarbonate activation of hydrogen peroxide: oxidation of sulfides by peroxymonocarbonate. J. Am. Chem. Soc..

[56] Davis AR, Oliver BG (1972). A vibrational-spectroscopic study of the species present in the CO_2_-H_2_O system. J. Solution Chem..

[57] GiguPrc PA, Lemaire D (1972). Spectroscopic study of the hydrogen peroxide derivatives and percarbonates KHCO_4_ and K_2_C_2_O_6_. Canad. J. Chem..

[58] Jones, D. P. & William P. Griffith. Alkali-metal Peroxocarbonates, M_2_[CO_3_]·nH_2_O_2_, M_2_[C_2_O_6_], M[HCO_4_]·nH_2_O, and Li_2_[CO_4_]H_2_O. *J. Chem. Soc., Dalton Trans*., 2526–2532, (1980).

[59] Siahrostami S, Li GL, Viswanathan V, Norskov JK (2017). One- or two-electron water oxidation, hydroxyl radical, or H_2_O_2_ evolution. J. Phys. Chem. Lett..

[60] Zhan S (2020). Efficient Fenton-like process for pollutant removal in electron-rich/poor reaction sites induced by surface oxygen vacancy over cobalt-zinc oxides. Environ. Sci. Technol..

[61] Hou X (2017). Hydroxylamine promoted goethite surface fenton degradation of organic pollutants. Environ. Sci Technol..

[62] Gill TM, Vallez L, Zheng X (2021). Enhancing electrochemical water oxidation toward H_2_O_2_ via carbonaceous electrolyte engineering. ACS Appl. Energy Mater..

[63] Wang G (2018). Removal of norfloxacin by surface Fenton system (MnFe_2_O_4_/H_2_O_2_): Kinetics, mechanism and degradation pathway. Chem. Eng. J..

[64] Rossmeisl J, Qu ZW, Zhu H, Kroes GJ, Nørskov JK (2007). Electrolysis of water on oxide surfaces. J. Electroanal. Chem..

[65] Burke MS, Enman LJ, Batchellor AS, Zou S, Boettcher SW (2015). Oxygen evolution reaction electrocatalysis on transition metal oxides and (Oxy)hydroxides: Activity trends and design principles. Chem. Mater..

[66] Man IC (2011). Universality in oxygen evolution electrocatalysis on oxide surfaces. ChemCatChem.

[67] Hong WT (2015). Toward the rational design of non-precious transition metal oxides for oxygen electrocatalysis. Energy Environ. Sci..

[68] Xinnian Z (2009). Photocatalytic decomposition of benzene by porous nanocrystalline ZnGa_2_O_4_ with a high surface area. Environ. Sci. Technol..

[69] Yan SC (2010). A room-temperature reactive-template route to mesoporous ZnGa_2_O_4_ with improved photocatalytic activity in reduction of CO2. Angew. Chem. Int. Ed. Engl..

[70] Kumagai N, Ni L, Irie H (2011). A visible-light-sensitive water splitting photocatalyst composed of Rh^3+^ in a 4d^6^ electronic configuration, Rh^3+^-doped ZnGa_2_O_4_. Chem. Commun..

[71] Klassen NV, Marchlngton D, McGowan HCE (1994). H_2_O_2_ determination by the I_3_^-^ method and by KMnO_4_ titration. Anal. Chem..

[72] Hu Z (2020). Cu(In,Ga)Se_2_ for selective and efficient photoelectrochemical conversion of CO_2_ into CO. J. Catal..

[73] Bachhuber F (2015). Van der Waals interactions in selected allotropes of phosphorus. Z. Kristallogr. - Cryst. Mater..

[74] Lee, J. et al. Electrochemical behavior of the flower-shaped CoMn_2_O_4_ spinel structure assembled for effective HER from water splitting. *Electrochim. Acta* **379**, 138168 (2021).

[75] Li A (2022). Enhancing the stability of cobalt spinel oxide towards sustainable oxygen evolution in acid. Nat. Catal..

[76] Liu Q (2022). Ambient ammonia synthesis via electrochemical reduction of nitrate enabled by NiCo_2_O_4_ nanowire array. Small.

[77] Jiao Y, Zheng Y, Jaroniec M, Qiao SZ (2014). Origin of the electrocatalytic oxygen reduction activity of graphene-based catalysts: a roadmap to achieve the best performance. J. Am. Chem. Soc..

[78] Gu Y (2018). Electronic structure tuning in Ni_3_FeN/r-GO aerogel toward bifunctional electrocatalyst for overall water splitting. ACS Nano.

[79] Duan H (2017). High-performance Rh_2_P electrocatalyst for efficient water splitting. J. Am. Chem. Soc..

[80] Zhang W, Xiao Y (2020). Mechanism of electrocatalytically active precious metal (Ni, Pd, Pt, and Ru) complexes in the graphene basal plane for ORR applications in novel fuel cells. Energy Fuels.

