# Supplementary material for: Electrochemical generation of hydrogen peroxide from a zinc gallium oxide anode with dual active sites
Source: Nat Commun. 2023 Apr 5;14:1890. doi: 10.1038/s41467-023-37007-9 (PMC10076521; doi:10.1038/s41467-023-37007-9)
Supplement: Supplementary file 1 — Supplementary Information [file 41467_2023_37007_MOESM1_ESM.pdf]

Supporting Information

Electrochemical generation of hydrogen peroxide from a zinc gallium oxide anode with dual active sites

Lejing Li,[1] Zhuofeng Hu,[2,*] Yongqiang Kang,[3] Shiyu Cao,[4] Liangpang Xu,[1] Luo Yu,[1] Lizhi Zhang,[4,*] Jimmy C. Yu[1,*]

[1] Department of Chemistry, The Chinese University of Hong Kong, Hong Kong SAR, China

[2] School of Environmental Science and Engineering
Guangdong Provincial Key Laboratory of Environmental Pollution Control and Remediation Technology, Sun Yat-sen University
Guangzhou 510275, China

[3] Institute of Materials Research, Tsinghua Shenzhen International Graduate School, Tsinghua University, Shenzhen 518055, China

[4] Key Laboratory of Pesticide & Chemical Biology of Ministry of Education, Institute of Applied & Environmental Chemistry,
College of Chemistry, Central China Normal University,
Wuhan, 430079 China

Corresponding Authors:
huzhf8@mail.sysu.edu.cn
zhanglz@mail.ccnu.edu.cn
jimyu@cuhk.edu.hk

Supplementary Tables

Table S1 Summary of H_2O_2 concentration plateau for different anode materials

Catalyst	Electrolyte; volume	H_2O_2 saturation level	Time/ Charge	Experimental conditions	Ref
$BiVO_4$	0.1 M $KHCO_3$; 35 mL	< 50 μM	3.6 coulombs	3.0 V vs. Ag/AgCl; CO_2 gas bubbling in an ice bath (below 5 °C)	[1]
$BiVO_4$	2 M $KHCO_3$; 35 mL	~5000 μM	220 coulombs	Applied voltage of 7.0 V; CO_2 gas bubbling in an ice bath (below 5 °C)	[1]
$CaSnO_3$	2 M $KHCO_3$; 30 mL	~30 ppm	12 h	2.2 V vs. RHE	[2]
$CaSnO_3$	2 M $KHCO_3$	~15 ppm<br>~20 ppm<br>~30 ppm	2 h<br>2 h<br>2 h	2.2 V vs. Ag/AgCl<br>2.4 V vs. Ag/AgCl<br>2.6 V vs. Ag/AgCl	[3]
Boron-doped diamond	1 M $HClO_4$	~ 0.9 mM	5 h	160 mA cm^{-2}	[4]
Boron-doped diamond	1 M $HClO_4$	~ 0.9 mM	2 h	1600 A m^{-2}	[5]
Boron-doped diamond	1 M $KHCO_3$	~10 mM	3500 s	3.17 V vs. RHE	[6]
Boron-doped diamond	2 M $KHCO_3$	~25 mM	2000 s	3.17 V vs. RHE	[6]
C,N codoped TiO_2	0.05 M Na_2SO_4 (pH was adjusted to 3 by H_2SO_4)	1.8 mM	6 h	2.9 V vs. Ag/AgCl	[7]
Sb_2O_3	2 M $KHCO_3$; 30 mL	~100 ppm	6 h	3.08 V vs. RHE	[8]
$CaSnO_3$	2 M $KHCO_3$; 30 mL	450 μmol	300 min	2.9 V vs. RHE	[9]
$ZnGa_2O_4$	2 M $KHCO_3$; 50 mL	~22 mM	180 min	2.7 V vs. RHE	This work
$ZnGa_2O_4$	2 M K_2CO_3; 60 mL	~54 mM	150 min	2.9 V vs. RHE	This work

Table S2 Summary of current density, H_2O_2 concentration and electrolysis time

**Potential (V vs. RHE)**	**Current density (mA cm^{-2})**	**Quantity of electric charge (Coulomb)**	**H_2O_2 (mM)**	**Time (s)**	**FE (%)**	**Productivity (µmol cm^{-2} min^{-1})**
2.0	2.8	2	0.39	713	38	0.33
2.1	6.6	3	0.76	455	49	1.01
2.2	15	3	0.93	205	60	2.72
2.3	25	3	1.28	122	82	6.27
2.5	42	5	1.76	119	68	8.96
2.7	69	5	1.58	73	61	13.05
2.9	118	5	1.27	42	49	17.91
3.1	162	5	1.09	32	42	21.23

Table S3 Summary of anodic H_2O_2 generation performance of different anode materials

Catalyst	Electrolyte	[a]Potential (V versus RHE)	FE (%)	Current density ($mA\ cm^{-2}$)	Maximum H_2O_2 generation rate	Ref
$BiVO_4$	1 M $NaHCO_3$	3.1	70	-	5.77 $\mu mol\ cm^{-2}\ min^{-1}$	10
$BiVO_4$:6%Gd	2 M $KHCO_3$	3.1	78	-	10.6 $\mu mol\ cm^{-2}\ min^{-1}$	11
$CaSnO_3$	2 M $KHCO_3$	3.2	76	~6 $mA\ cm^{-2}$ at 2.8 V vs. RHE	4.6 $\mu mol\ cm^{-2}\ min^{-1}$	2
ZnO	2 M $KHCO_3$	2.6	81	~ 10 $mA\ cm^{-2}$ at 2.6 V vs. RHE	[b]4.15 $\mu mol\ cm^{-2}\ min^{-1}$	12
Sb_2O_3	2 M $KHCO_3$	3.08	22	~ 3.5 $mA\ cm^{-2}$ at 3.08 V vs. RHE	0.26 $\mu mol\ cm^{-2}\ min^{-1}$	8
Bi_2WO_6:5%Mo	2 M $KHCO_3$	3.2	79	~ 10 $mA\ cm^{-2}$ at 3.2 V vs. RHE	5 $\mu mol\ cm^{-2}\ min^{-1}$	13
$CuWO_4$:Sn	2 M $KHCO_3$	2.5	72	~ 32 $mA\ cm^{-2}$ at 2.5 V vs. RHE	11.6 $\mu mol\ cm^{-2}\ min^{-1}$	14
BDD	2 M $KHCO_3$	3.17	28	~ 120 $mA\ cm^{-2}$ at 3.17 V vs. RHE	19.7 $\mu mol\ cm^{-2}\ min^{-1}$	6
BDD	2 M $K_2CO_3/KHCO_3$ (salt ratios 1:1)	2.85	87	~ 200 $mA\ cm^{-2}$ at 3.0 V vs. RHE	76.4 $\mu mol\ cm^{-2}\ min^{-1}$	15
CFP-60%PTFE	1 M Na_2CO_3	2.4	66	~ 100 $mA\ cm^{-2}$ at 2.4 V vs. RHE	23.4 $\mu mol\ cm^{-2}\ min^{-1}$	16
$ZnGa_2O_4$	2 M $KHCO_3$	2.3	82	~ 25 $mA\ cm^{-2}$ at 2.3 V vs. RHE	22.5 $\mu mol\ cm^{-2}\ min^{-1}$	This work
$ZnGa_2O_4$	2 M K_2CO_3	2.9	77	~ 242 $mA\ cm^{-2}$ at 2.9 V vs. RHE	69 $\mu mol\ cm^{-2}\ min^{-1}$	This work

[a]NB: The potential corresponding to the highest FE. [b]NB: H_2O_2 production rate is calculated based on the values of jH_2O_2 reported.

Table S4 Summary of adsorption energy of bicarbonate/percarbonate on different models

	**$ZnGa_2O_4$ (311)**	**ZnO (200)**	**Ga_2O_3 (020)**
$*HCO_3$	-1.9 eV	-0.3 eV	-2.6 eV
$*HCO_4$	-2.9 eV	-1.1 eV	-1.8 eV

Supplementary Figures

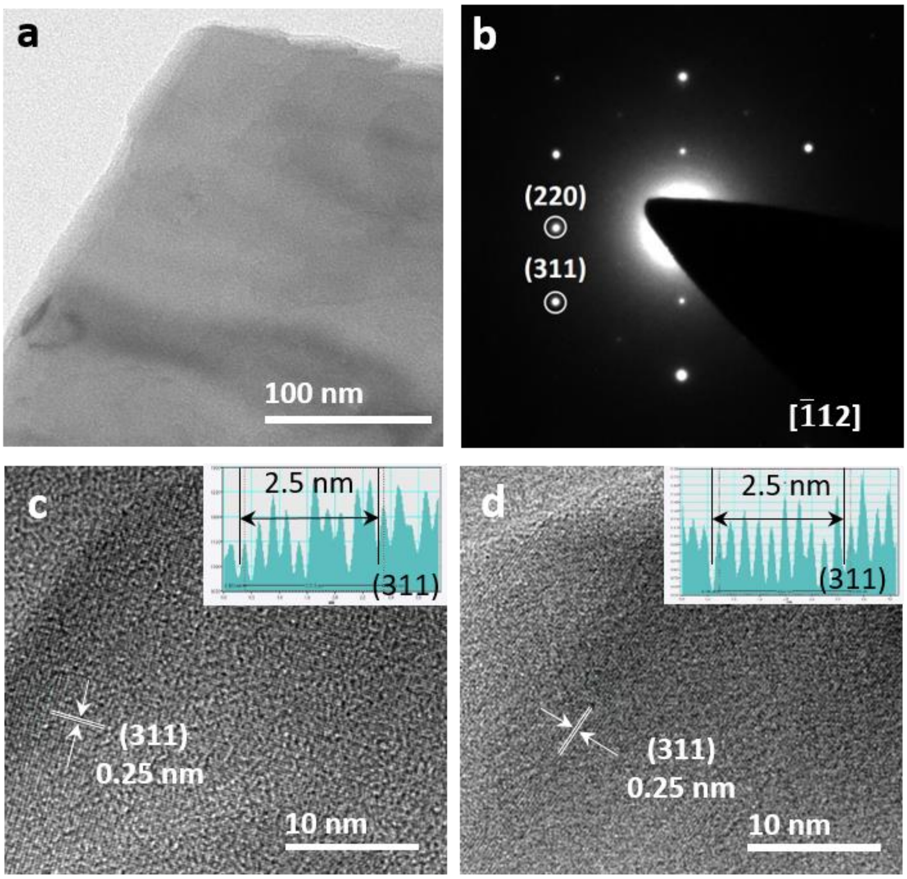

Figure S1. **Microscopic informantion of $ZnGa_2O_4$ catalyst**. (a) TEM image of $ZnGa_2O_4$ catalyst. (b) Selected area electron diffraction pattern and (c, d) HRTEM images.

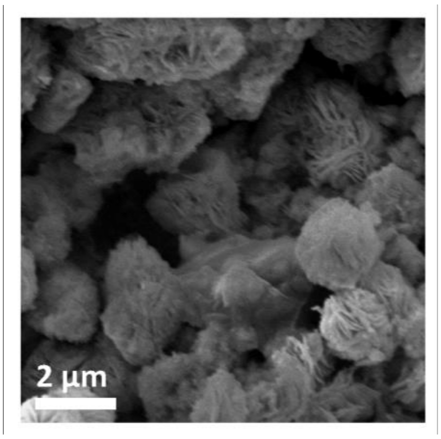

Figure S2. **The SEM images of the as-prepared $ZnGa_2O_4$ anode surface**.

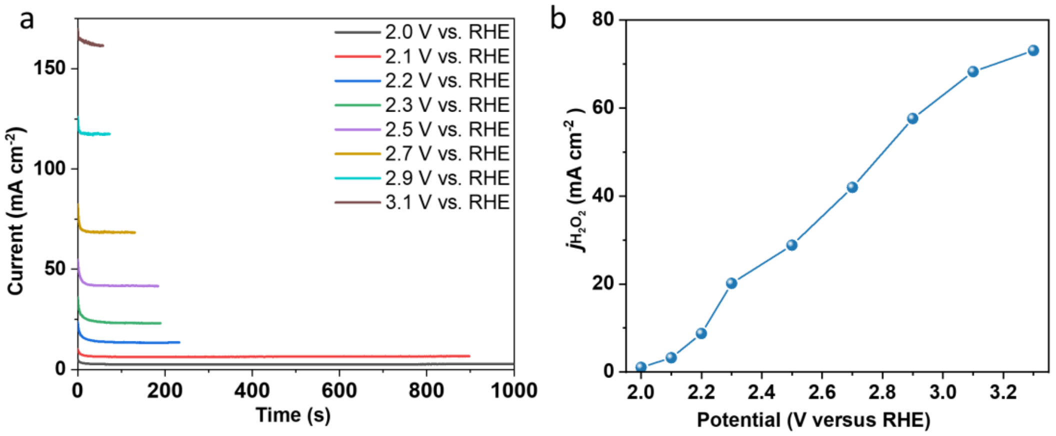

Figure S3. **The I-T curves of $ZnGa_2O_4$ anode at different applied potentials (a) and the H_2O_2 generation partial current density versus applied potential on $ZnGa_2O_4$ anode (b)**.

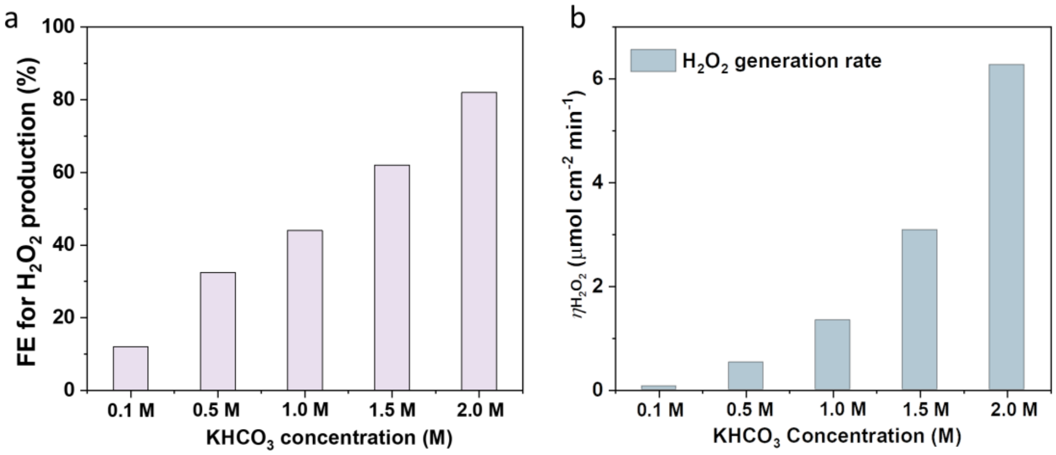

Figure S4. **The effect of $KHCO_3$ concentration on the H_2O_2 generation performance of $ZnGa_2O_4$ anode**. The (a) H_2O_2 FE and (b) generation rate of $ZnGa_2O_4$ anode in $KHCO_3$ electrolytes with different concentrations.

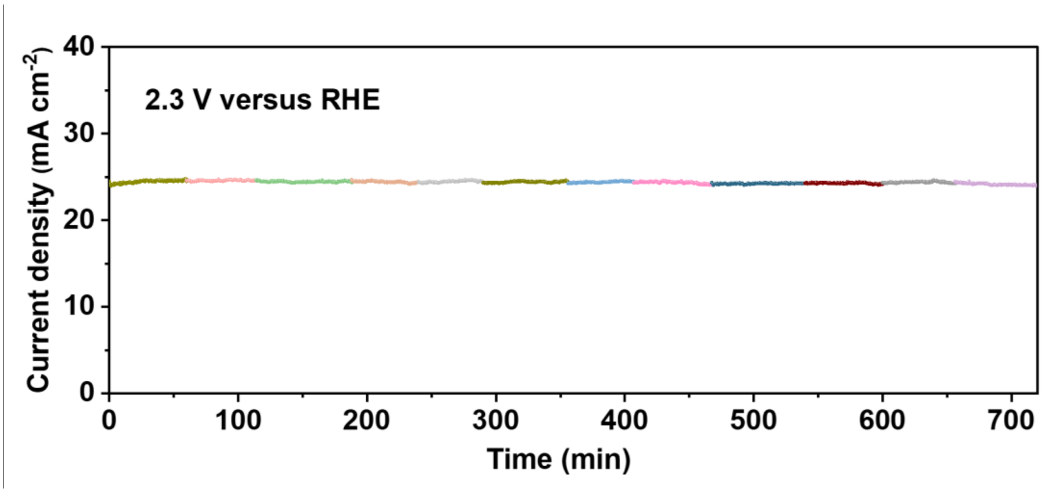

Figure S5. The current density of $ZnGa_2O_4$ anode at an potential of 2.3 V versus RHE in 2 M $KHCO_3$ during continuous test cycles.

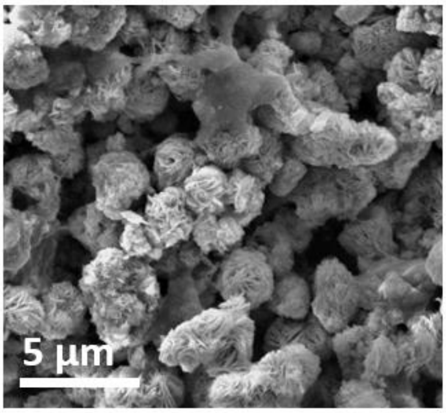

Figure S6. SEM images of the $ZnGa_2O_4$ anode after the stability test.

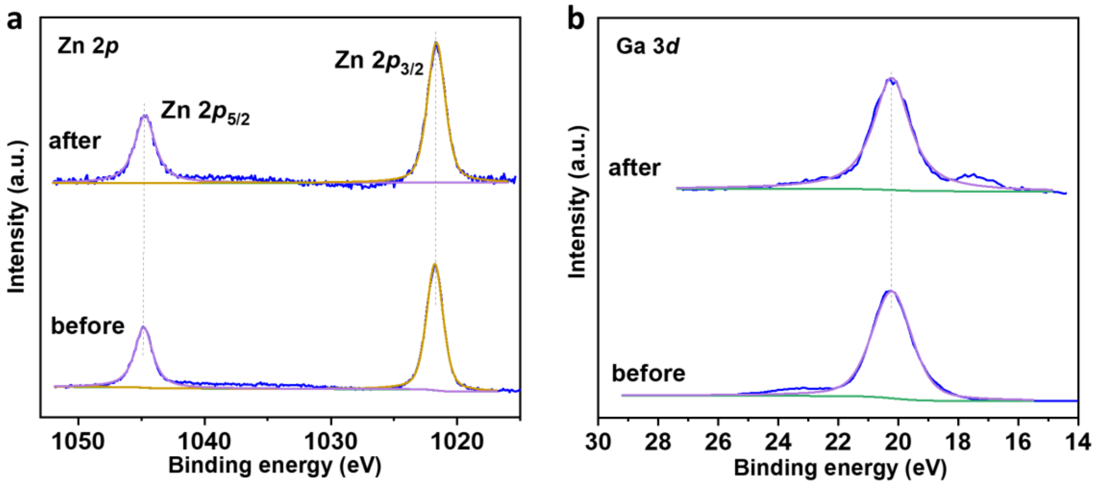

Figure S7. XPS characterizations of $ZnGa_2O_4$. High-resolution Zn 2*p* (a) and Ga 3*d* (b) X-ray photoelectron spectroscopy spectra of $ZnGa_2O_4$ before and after the stability test.

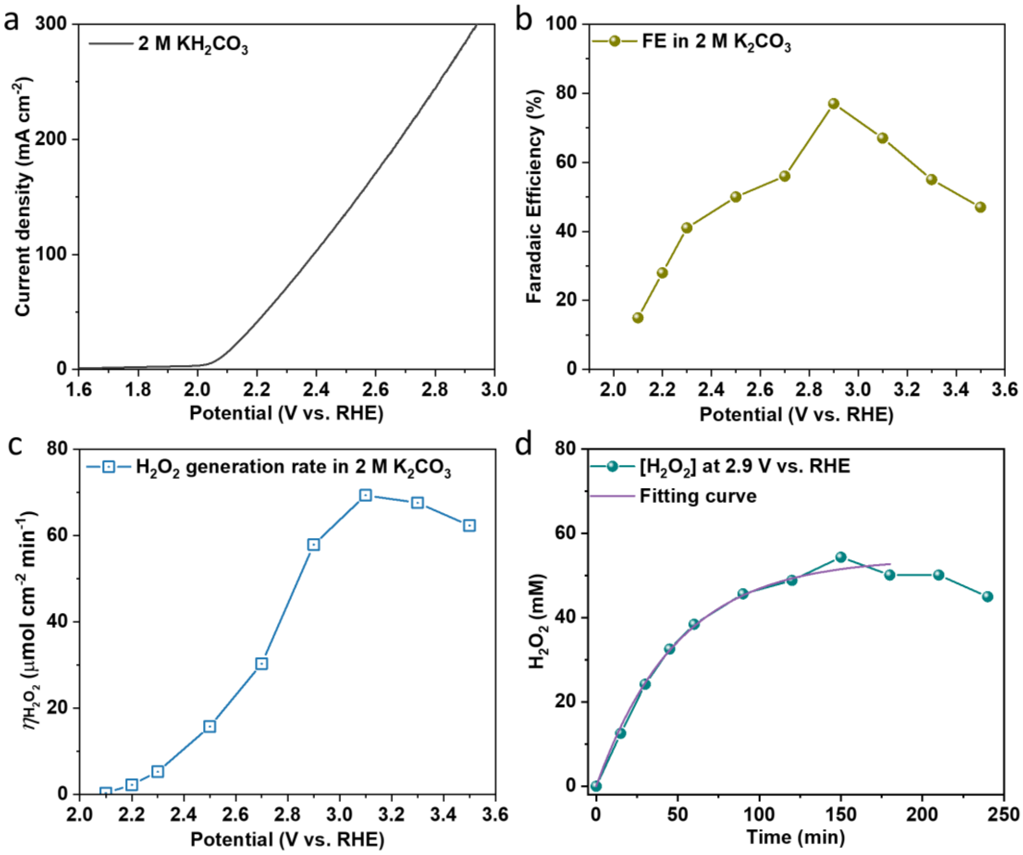

Figure S8. H_2O_2 generation performance on $ZnGa_2O_4$ anode in 2 M K_2CO_3 solution. (a) LSV curve of $ZnGa_2O_4$ anode recorded in 2 M K_2CO_3. The scan rate is 5 mV s^{-1}. (b) H_2O_2 FE of $ZnGa_2O_4$ anode at different potentials in 2 M K_2CO_3 electrolyte. (c) The H_2O_2 generation rate on $ZnGa_2O_4$ anode at different potentials. (d) The accumulated H_2O_2 concentration in 4 h-electrolysis at 2.9 V versus RHE. (60 mL electrolyte, electrode area = 0.5 cm^2).

There are several reaction pathways have been proposed for H_2O_2 generation in carbonate-based solutions. A hydroxyl radical-mediated reaction pathway has been reported on a boron-doped diamond anode in highly concentrated carbonate electrolytes, and direct experimental evidence and theoretical calculations have also been demonstrated.[15,17,18] For example, Ruiz et. al reported that hydroxyl radicals formed on the BDD surface can act as an intermediate in the oxidation of bicarbonate ions in the supporting electrolyte into $C_2O_6^{2-}$.[25] Recently, the report from Mavrikis et. al reveal the positive effect of CO_3^{2-} anions on the anodic H_2O_2 generation on BDD. They proposed that hydroxyl radicals can react with carbonate anions to give radicals ($CO_3^{\cdot-}$), and a further oxidation of carbonate radical would give peroxymonocarbonate (HCO_4^-).[18] Moreover, based on a direct relationship between CO_3^{2-} ion activity and enhanced H_2O_2 generation performance, Dhananjai et. al also suggested similar reaction pathway

(peroxodicarbonate species ($C_2O_6^{2-}$) can be formed by oxidizing CO_3^{2-} species) on the carbon fiber paper anode.[19] Therefore, it is inferred that the reaction pathway varies on different anode materials even in similar carbonate/bicarbonate solution.

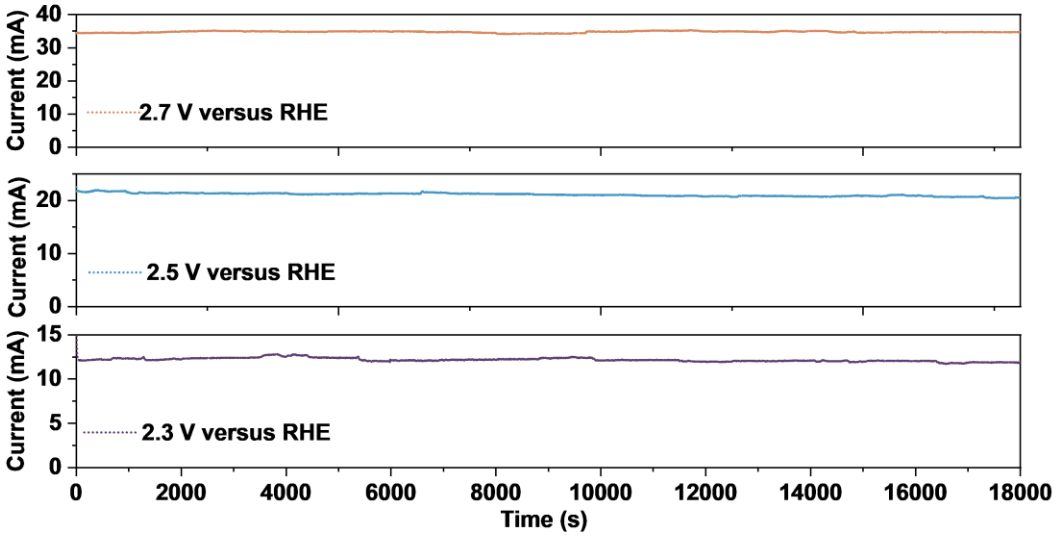

Figure S9. **The I-T curves of $ZnGa_2O_4$ in 300-min electrolysis at different potentials**.

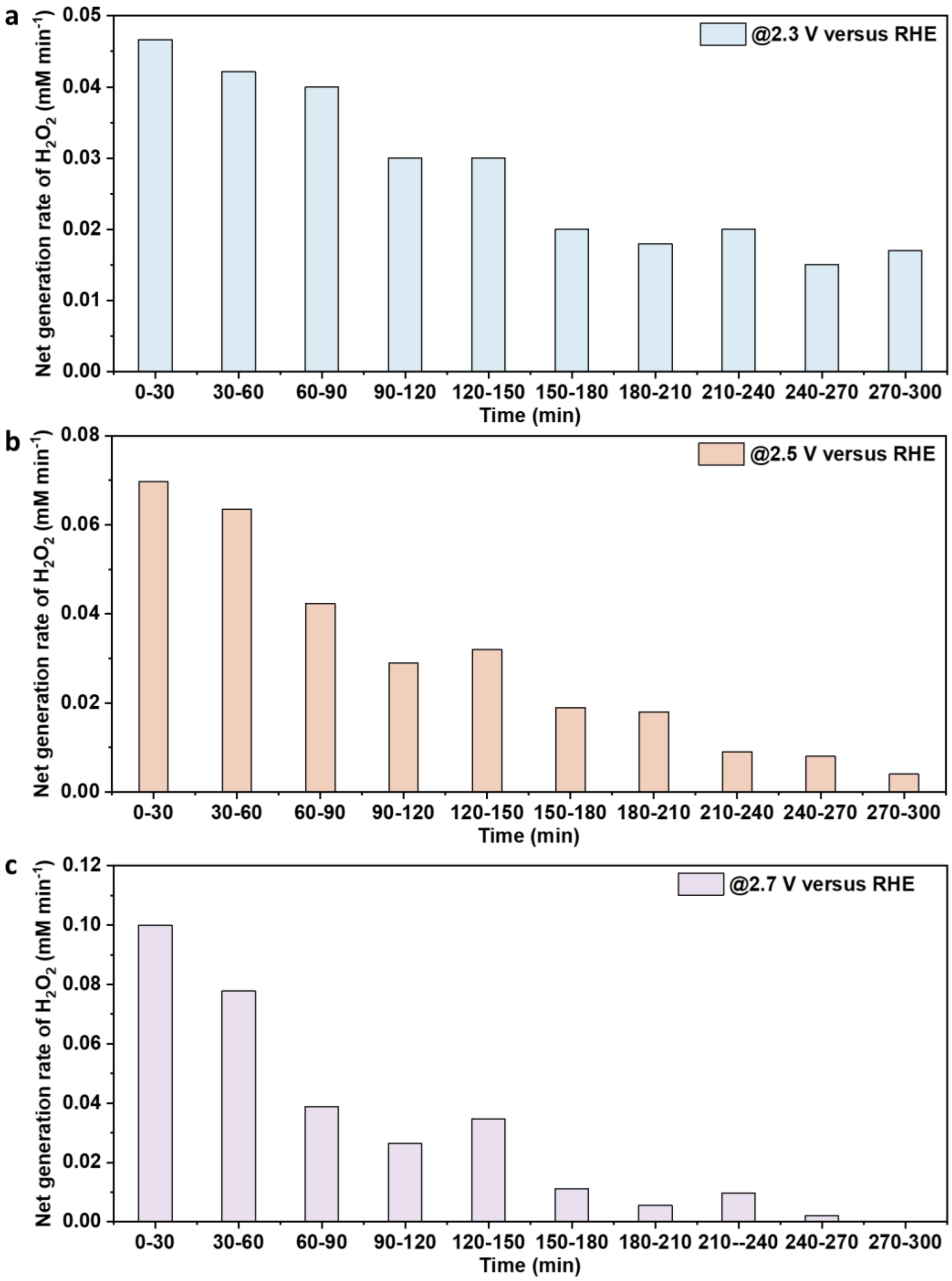

Figure S10. **The apparent generation rate of H_2O_2 as a function of time at different potentials.** H_2O_2 gneration rate at 2.3 V versus RHE (a), 2.5 V versus RHE (b) and 2.7 V versus RHE (c).

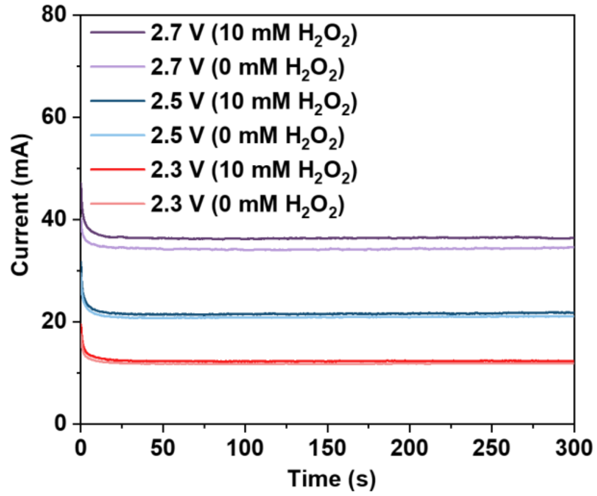

Figure S11. The chronoamperometric data for the estimation of H_2O_2 electrodecomposition on $ZnGa_2O_4$ anode in 2 M $KHCO_3$ with and without manual addition of H_2O_2.

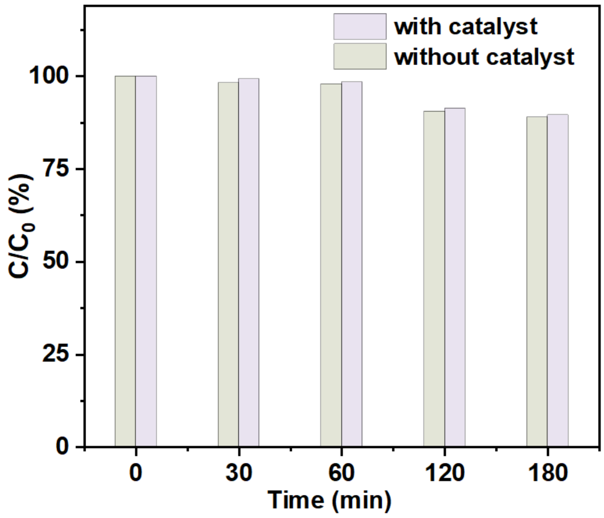

Figure S12. Self-decomposition of H_2O_2 in 2 M $KHCO_3$ solution with and without the presence of $ZnGa_2O_4$ powder catalyst (500 mg L^{-1}). Experimental conditions: [H_2O_2] = 2 mM; in dark; 20 °C.

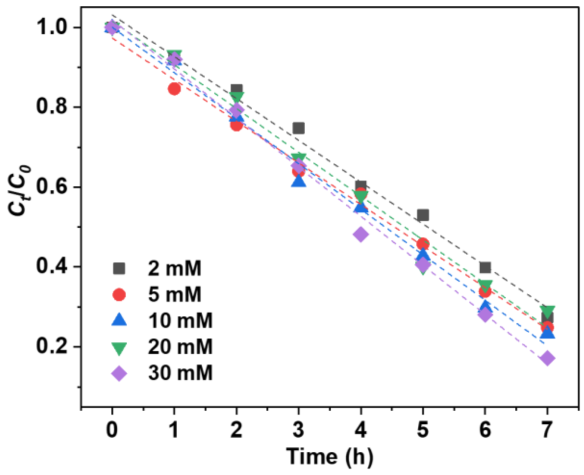

Figure S13. **The relationship between the $[C_t/C_0]$ and time**. The linear fitting curves indicate that the disproportionation of H_2O_2 in 2 M $KHCO_3$ solution is zero-order reaction when the initials concentration of H_2O_2 is in the range of 2 ~ 30 mM.

The H_2O_2 decomposition rate constant k was calculated according to the following equation: $[C_t/C_0] = -k \times t$ (1)

C_t is the H_2O_2 concentration after any given time t, C_0 is the initial concentration of H_2O_2. The calculated decomposition rate constants are 0.10, 0.10, 0.11, 0.11, 0.12 h^{-1} at C_0 of 2, 5, 10, 20, 30 mM.

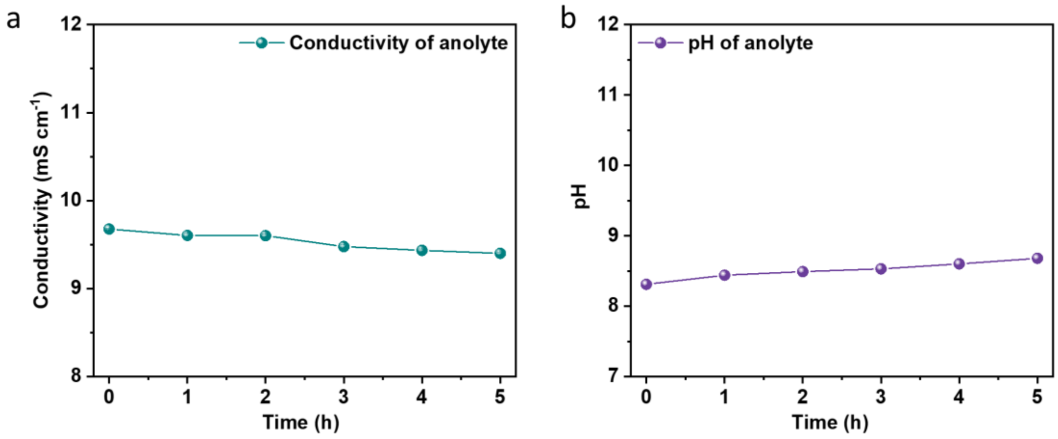

Figure S14. **The corresponding changes in conductivity (a) and pH (b) of the anolyte over time at a potential of 2.3 V vs. RHE**. This experiment was carried out in an H-cell in which the anolye and catholyte is devided by a Nafion 117 membrane. 2 M $KHCO_3$ was used as both anolyte and catholyte. The anode area is 0.5 cm^2. A Pt foil (1 cm^2) was uased as the counter electrode. Before the conductivity test, 0.5 mL of anolyte was diluted into 9.5 mL DI water. The

electro-oxidtion of water would cause pH decrease during continuous electrolysis. Meanwhile, the hydrogen evolution reaction takes place at the cathode side, which leads to increased pH of the catholyte (increase from 8.31 to 9.12 after 5 h-electrolysis). Thus the increased pH of anolyte from 8.31 to 8.68 should be related to the proton diffusion through the Nafion membrane.

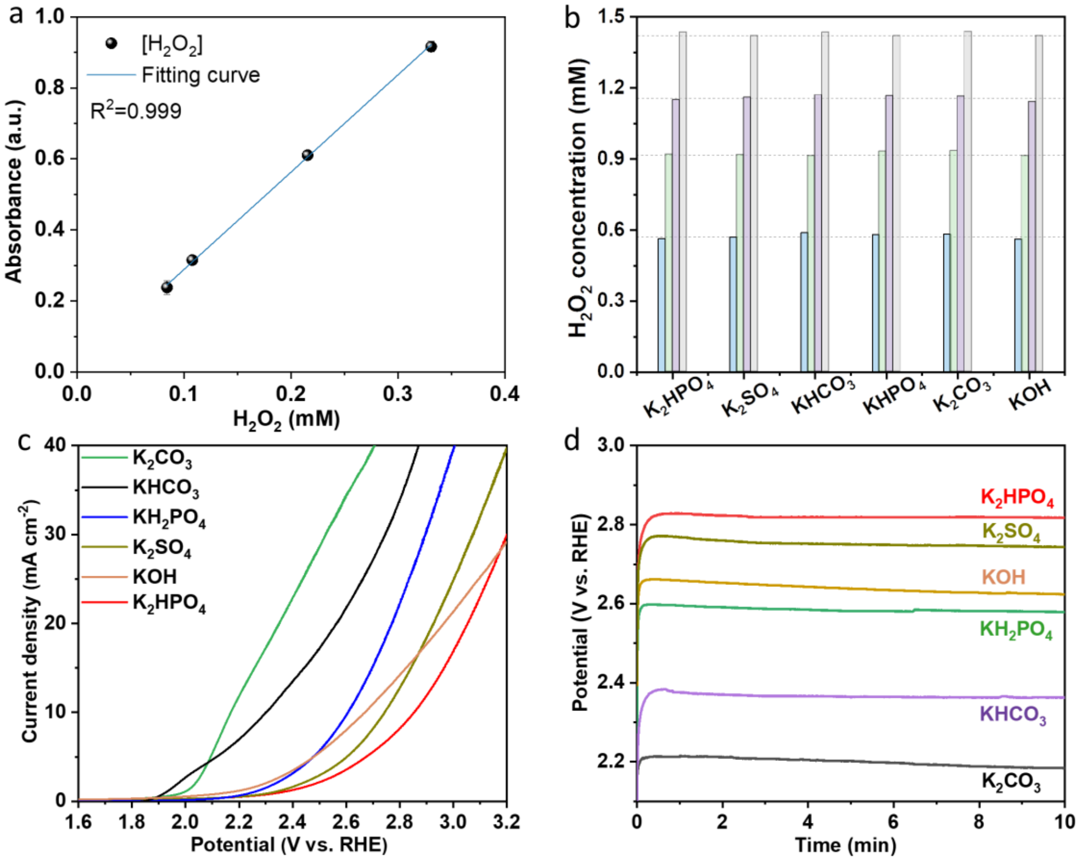

Figure S15. **H_2O_2 generation performance of $ZnGa_2O_4$ anode in different electrolytes**. (a) The calibration curve of H_2O_2 in DI water based on the I_3^- method. (b) Determination of H_2O_2 concentration in different electrolyte (0.5 M) by the I_3^- method. The pH of the electrolytes containing H_2O_2 were adjusted to 7 before test. (c) The LSV curves of $ZnGa_2O_4$ anode in different electrolytes (0.5 M). (d) Chronopotentiometry of $ZnGa_2O_4$ anode at 8 mA cm^{-2} in different electrolytes (0.5 M).

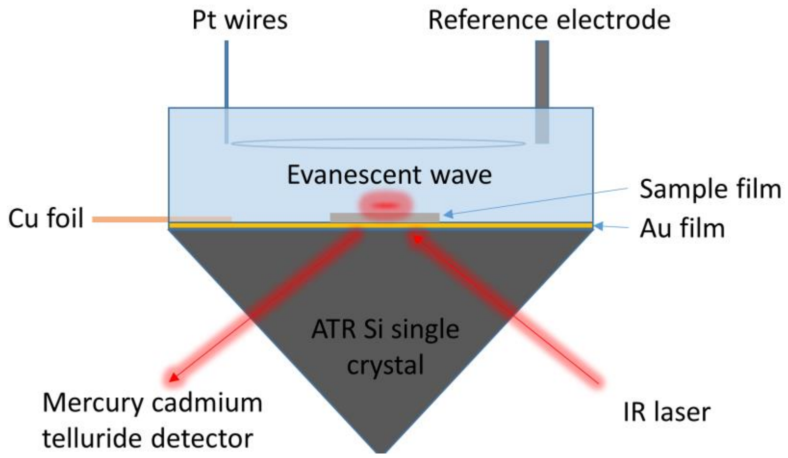

Figure S16. **Schematic representation of the in situ electrochemical cell for the ATR-FTIR test.**

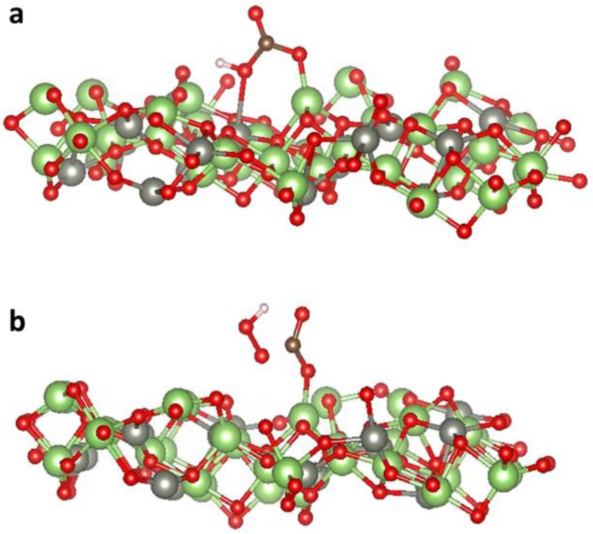

Figure S17. Models of (a) HCO_3-$ZnGa_2O_4$ and (b) HCO_4-$ZnGa_2O_4$ at (220) facet. The HCO_3^- adsorbed on the (220) facet of $ZnGa_2O_4$ by forming a bridged adsorption configuration (two O atoms of $^*HCO_3$ binding with one Zn atom and one Ga atom).

In the case of the ($\bar{1}$12) facet, the E_{*HCO4} - E_{*HCO3} is 0.43 eV; E_H is -3.39 eV; E_{OH} is -7.34 eV; U is 2.3 V. Therefore, ΔE is calculated to be -0.22 eV (E_{*HCO4} - E_{*HCO3} + E_H - E_{OH}- 2eU).

The ZPE for HCO_3 is 0.66 eV; ZPE for HCO_4 is 0.80 eV; ZPE for H is 0.16 eV; ZPE for OH is 0.33 eV; ΔZPE is -0.03 eV (0.80 eV- 0.66 eV + 0.16 eV - 0.33 eV = 0.03 eV). At 298 K, TS for HCO_3 is 0.05 eV; TS for HCO_4 is 0.14 eV; TS for H is 0.13 eV; TS for OH is 0.07 eV;

therefore, TΔS is 0.15 eV (0.14 eV - 0.05 eV + 0.13 eV - 0.07 eV = 0.15 eV). Therefore, ΔZPE - TΔS is -0.18 eV, and ΔG is calculated to be -0.39 eV at the potential of 2.3 V versus RHE at 298 K. This negative value suggests that at 2.3 V versus RHE the transformation of $*HCO_3$ to $*HCO_4$ on ($\bar{1}12$) facet is favorable.

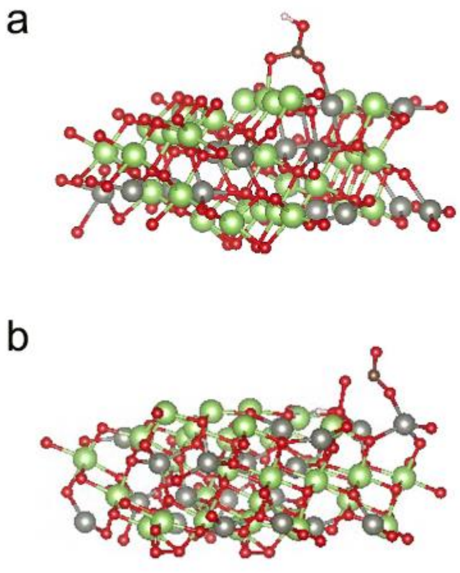

Figure S18. Models of (a) HCO_3-$ZnGa_2O_4$ and (b) HCO_4-$ZnGa_2O_4$ at ($\bar{1}12$) facet. The HCO_3^- adsorbed on the ($\bar{1}12$) facet of $ZnGa_2O_4$ by forming a bridged adsorption configuration (two O atoms of $*HCO_3$ binding with one Zn atom and one Ga atom).

Given that temperature can affect the value of ΔG by TΔS, then S and the corresponding TΔS at different temperatures were calculated (**Table S5**). It can be found that the value of TΔS increases from 0.10 eV at 200 K to 0.37 eV at 700 K. This means that the value of ΔG will become more negative when temperature increases, indicating the reaction is easier to occur at higher temperatures. Actually, compared with the value of ΔG (-1.82 eV), the change of TΔS is relatively small, which will not greatly affect the result.

Table S5. Value of TΔS at different temperatures

T (K)	S for HCO_4 ($J\ K^{-1}mol^{-1}$)	S for HCO_3 ($J\ K^{-1}mol^{-1}$)	S for H ($J\ K^{-1}mol^{-1}$)	S for OH ($J\ K^{-1}mol^{-1}$)	ΔS ($J\ K^{-1}\ mol^{-1}$)	TΔS (eV)
200	32.55562	7.823424	38.16827935	15.6007027	47.29978	0.098214
250	39.30174	12.5424	40.0234601	19.013863	47.76894	0.123985

300	45.96567	17.52045	41.53929155	21.8813082	48.10321	0.149823
350	52.48186	22.55981	42.82092825	24.3446144	48.39837	0.175866
400	58.80062	27.54415	43.9311494	26.4998665	48.68775	0.202192
450	64.89147	32.40514	44.9104706	28.4139118	48.98289	0.228844
500	70.73955	37.10485	45.7866001	30.1348302	49.28647	0.255848
550	76.34121	41.62491	46.5793742	31.6983507	49.59732	0.283207
600	81.70033	45.95941	47.3035347	33.1317966	49.91265	0.310918
650	86.82552	50.11015	47.97038135	34.4565538	50.22919	0.338964
700	91.72812	54.08342	48.5887958	35.6896561	50.54384	0.367324

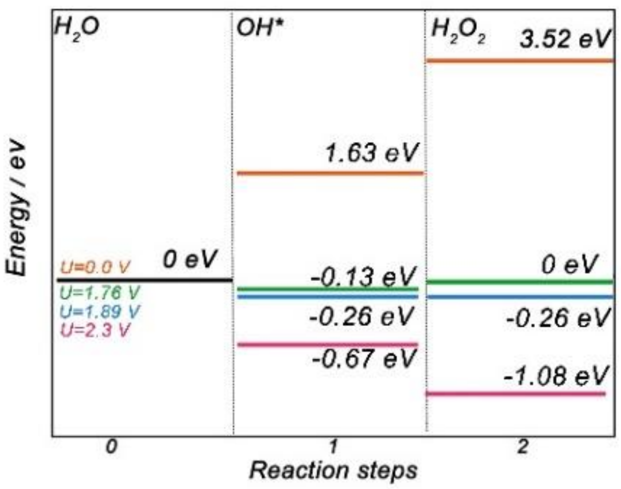

Figure S19. **Energy diagram for H_2O_2 evolution through direct pathway on $ZnGa_2O_4$.** The theoretical equilibrium potential for H_2O_2 production *via* water oxidation is 1.76 V versus RHE. Therefore, in the first step of *OH adsorption, the potential should be larger than 1.76 V versus RHE, and the second step requires a potential of 3.52 eV because it is a two-electron process. To produce H_2O_2, the energy needs to fall from the first step to the second step. For $ZnGa_2O_4$, the ΔG_{OH*} is calculated to be 1.63 eV. Therefore, at the theoretical potential of 1.76 V, although the energy at the first step is -0.13 eV, the energy is 0 eV for the second step (**Figure S19**). As a result, it is still unfavorable for H_2O_2 generation because the energy needs to go up from -0.13 eV to 0 eV at the second step. The bias needs to be larger than1.89 V so that both the first and second steps are energy favorable for H_2O_2 generation. Therefore, for $ZnGa_2O_4$, based on the DFT results the lowest potential to produce H_2O_2 is 1.89 V, which is very close to our experimental value of 2.0 eV. At a larger bias of 2.3 V, the energy diagram keeps going down

from -0.67 eV to -1.08 eV (**Figure S19**), suggesting H_2O_2 can be easily generated at this bias.

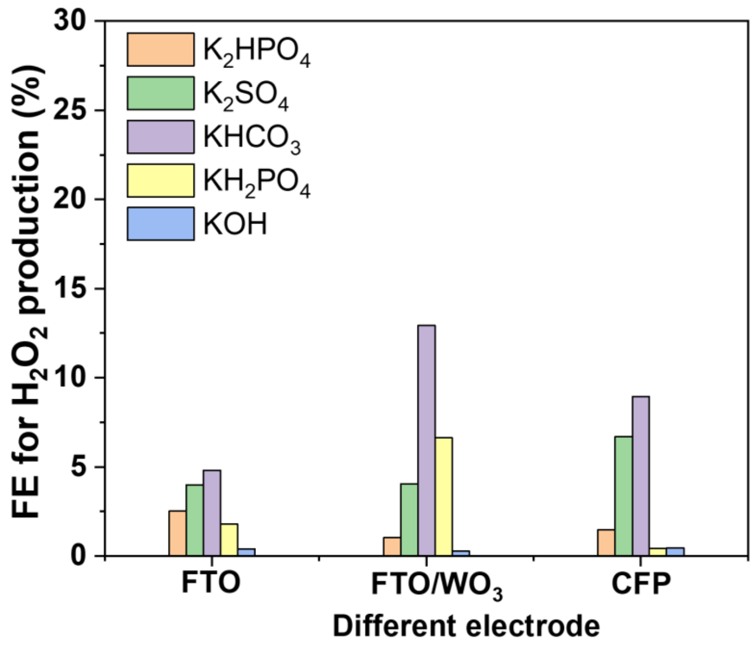

Figure S20. The FE of anodic H_2O_2 generation of anodes including F-doped tin oxide (FTO), FTO/WO_3. Toray-h-060 carbon fiber paper (CFP) at 3.0 V versus RHE in different electrolytes (0.5 M).

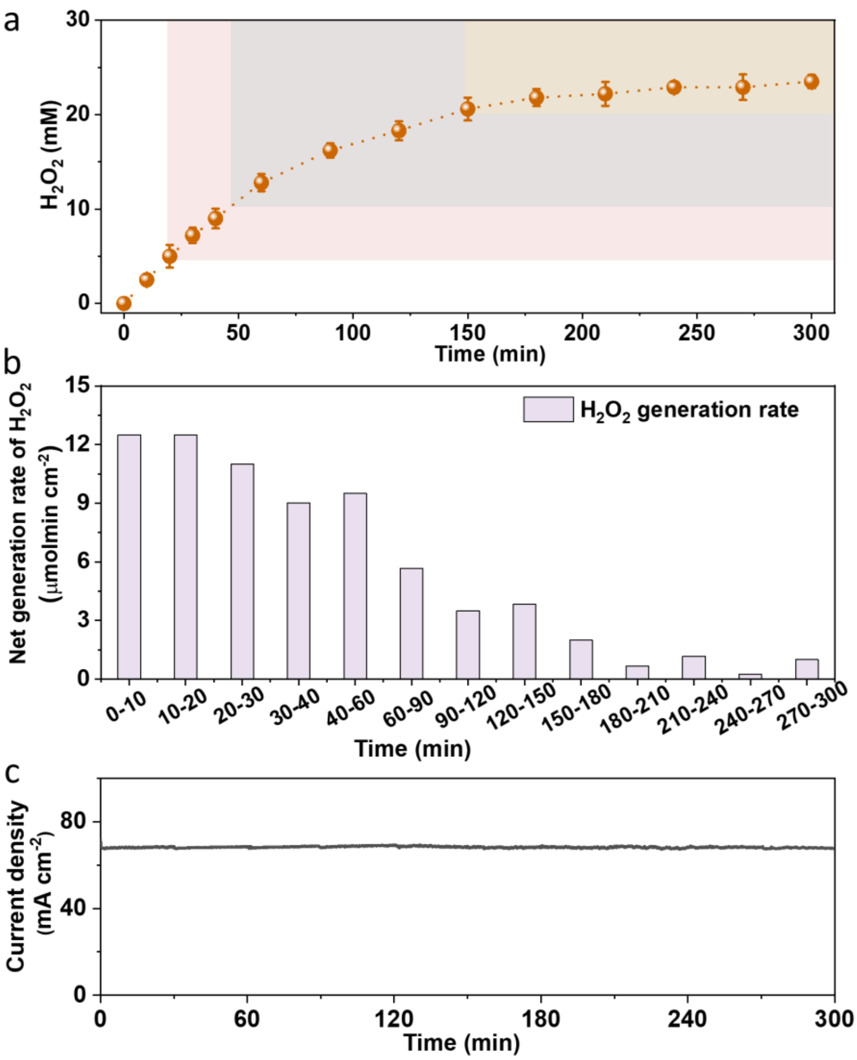

Figure S21. H_2O_2 generation performance of $ZnGa_2O_4$ anode at 2.7 V versus RHE in 300 min. (a) The real-time concentration of H_2O_2 at 2.7 V versus RHE. (b) The apparent generation rate of H_2O_2 as a function of time. (c) The recorded current profile of $ZnGa_2O_4$ anode at a potential of 2.7 V versus RHE. Experimental conditions: anode area: 1 cm^2; electrolyte: 50 mL 2 M $KHCO_3$.

Reference:

1 Fuku, K., Miyase, Y., Miseki, Y., Gunji, T. & Sayama, K. Enhanced Oxidative Hydrogen Peroxide Production on Conducting Glass Anodes Modified with Metal Oxides. *Chemistryselect* **1**, 5721-5726, (2016).

2 Park, S. Y. *et al.* $CaSnO_3$: An Electrocatalyst for Two-electron Water Oxidation Reaction to Form H_2O_2. *ACS Energy Lett.* **4**, 352-357, (2018).

3 Kang, T. *et al.* Efficient Hydrogen Peroxide (H_2O_2) Synthesis by $CaSnO_3$ via Two-Electron Water Oxidation Reaction. *ACS Sustainable Chem. Eng.* **8**, 15005-15012, (2020).

4 Marselli, B., Garcia-Gomez, J., Michaud, P.-A., Rodrigo, M. A. & Comninellis, C. Electrogeneration of Hydroxyl Radicals on Boron-Doped Diamond Electrodes. *J. Electrochem.*

Soc. **150**, 79-83, (2003).

5 Michaud, P.-A. *et al.* Electrochemical oxidation of water on synthetic boron-doped diamond thin film anodes. *J. Appl. Electrochem.* **33**, 151–154, (2003).

6 Mavrikis, S., Göltz, M., Rosiwal, S., Wang, L. & Ponce de León, C. Boron-Doped Diamond Electrocatalyst for Enhanced Anodic H_2O_2 Production. *ACS Appl. Energy Mater.* **3**, 3169-3173, (2020).

7 Xue, S. G. *et al.* Selective Electrocatalytic Water Oxidation to Produce H_2O_2 Using a C,N Codoped TiO_2 Electrode in an Acidic Electrolyte. *ACS Appl. Mater. Interfaces* **12**, 4423-4431, (2020).

8 Wang, Y., Lian, X., Zhou, Y., Guo, W. & He, H. Synthesis and characterization of Sb_2O_3: a stable electrocatalyst for efficient H_2O_2 production and accumulation and effective degradation of dyes. *New J. Chem.* **45**, 8958-8964, (2021).

9 Zhang, C. *et al.* High Yield Electrosynthesis of Hydrogen Peroxide from Water Using Electrospun $CaSnO_3$@Carbon Fiber Membrane Catalysts with Abundant Oxygen Vacancy. *Adv. Func. Mater.* **31**, 2100099, (2021).

10 Shi, X. J. *et al.* Understanding activity trends in electrochemical water oxidation to form hydrogen peroxide. *Nat. Commun.* **8**, 701, (2017).

11 Baek, J. H. *et al.* Selective and Efficient Gd-Doped $BiVO_4$ Photoanode for Two-electron Water Oxidation to H_2O_2. *ACS Energy Lett.* **4**, 720-728, (2019).

12 Kelly, S. *et al.* ZnO as an Active and Selective Catalyst for Electrochemical Water Oxidation to Hydrogen Peroxide. *ACS Catal.* **9**, 4593-4599, (2019).

13 Li, L., Hu, Z. & Yu, J. C. On-Demand Synthesis of H_2O_2 by Water Oxidation for Sustainable Resource Production and Organic Pollutant Degradation. *Angew. Chem. Int. Ed. Engl.* **59**, 20538-20544, (2020).

14 Li, L. *et al.* Direct Hydrogen Peroxide Synthesis on a Sn-doped $CuWO_4$/Sn Anode and an Air-Breathing Cathode. *Chem. Mater.* **34**, 63–71, (2022).

15 Mavrikis, S. *et al.* Effective Hydrogen Peroxide Production from Electrochemical Water Oxidation. *ACS Energy Lett.* **6**, 2369-2377, (2021).

16 Xia, C. *et al.* Confined Local Oxygen Gas Promotes Electrochemical Water Oxidation to Hydrogen Peroxide. *Nat. Catal.* **3**, 125-134, (2020).

17 Ruiz, E. J., Ortega-Borges, R., Jurado, J. L., Chapman, T. W. & Meas, Y. Simultaneous Anodic and Cathodic Production of Sodium Percarbonate in Aqueous Solution. *Electrochem. Solid-State Lett.* **12**, E1-E4, (2009).

18 Mavrikis, S., Goltz, M., Rosiwal, S., Wang, L. & Ponce de Leon, C. Carbonate-Induced Electrosynthesis of Hydrogen Peroxide via Two-Electron Water Oxidation. *ChemSusChem* **15**, e202102137, (2022).

19 Pangotra, D. *et al.* Anodic production of hydrogen peroxide using commercial carbon materials. *Appl. Catal., B* **303**, (2022).
